# Prostate cancer-derived holoclones: a novel and effective model for evaluating cancer stemness

**DOI:** 10.1038/s41598-020-68187-9

**Published:** 2020-07-09

**Authors:** Louise Flynn, Martin P. Barr, Anne-Marie Baird, Paul Smyth, Orla M. Casey, Gordon Blackshields, John Greene, Stephen R. Pennington, Emily Hams, Padraic G. Fallon, John O’Leary, Orla Sheils, Stephen P. Finn

**Affiliations:** 1 Department of Histopathology and Morbid Anatomy, Trinity Translational Medicine Institute, Trinity College Dublin, Dublin, Ireland; 2 Thoracic Oncology Research Group, Trinity Translational Medicine Institute, St. James’s Hospital, Trinity College Dublin, Dublin, Ireland; 3 School of Medicine, University College Dublin, Conway Institute Belfield, Dublin, Ireland; 4 School of Medicine, Trinity Translational Medicine Institute, Trinity Biomedical Sciences Institute, Trinity College Dublin, Dublin, Ireland; 5 Molecular Pathology Laboratory, Coombe Women and Infant’s University Hospital, Dublin, Ireland; 6 Department of Histopathology, St. James’s Hospital, Dublin, Ireland

**Keywords:** Prostate cancer, Cancer stem cells

## Abstract

Prostate cancer accounts for approximately 13.5% of all newly diagnosed male cancer cases. Significant clinical burdens remain in terms of ineffective prognostication, with overtreatment of insignificant disease. Additionally, the pathobiology underlying disease heterogeneity remains poorly understood. As the role of cancer stem cells in the perpetuation of aggressive carcinoma is being substantiated by experimental evidence, it is crucially important to understand the molecular mechanisms, which regulate key features of cancer stem cells. We investigated two methods for in vitro cultivation of putative prostate cancer stem cells based on ‘high-salt agar’ and ‘monoclonal cultivation’. Data demonstrated ‘monoclonal cultivation’ as the superior method. We demonstrated that ‘holoclones’ expressed canonical stem markers, retained the exclusive ability to generate poorly differentiated tumours in NOD/SCID mice and possessed a unique mRNA-miRNA gene signature. miRNA:Target interactions analysis visualised potentially critical regulatory networks, which are dysregulated in prostate cancer holoclones. The characterisation of this tumorigenic population lays the groundwork for this model to be used in the identification of proteomic or small non-coding RNA therapeutic targets for the eradication of this critical cellular population. This is significant, as it provides a potential route to limit development of aggressive disease and thus improve survival rates.

## Introduction

Prostate cancer (PCa) represents a major cause of cancer-related mortality and morbidity in men. With the exception of lung cancer, PCa has an incidence higher than that of all other solid-organ malignancies^1^. The molecular pathology of PCa is complex and involves multiple genes and environmental factors. Older age, ethnicity and positive family history have long been recognised as significant etiological factors for the development of the disease^2^. PCa is an inherently heterogeneous disease, comprising of multiple phenotypically diverse cancer cell types and a varied genomic landscape^3,4^. Thus, it can range in clinical behaviour from indolent to a rapidly fatal, aggressive malignancy^3–5^. The intrinsically heterogeneous nature of this malignancy is one of the major confounding factors in not only understanding, but also successfully treating PCa. In recent years, the development of the cancer stem cell (CSC) hypothesis has catalysed a paradigm shift in our understanding of the biologic mechanisms underlying malignancy^6^, particularly in PCa^7,8^.

Stem cells have long been known to occur in somatic tissues which undergo rapid regeneration including bone marrow and the skin^9^. However, in recent years the presence of stem cells has been recognised in more quiescent tissues, such as the prostate. In fact, the maintenance of the prostate gland structure and function is dependent upon a repository of multipotent and stromal stem cells residing within the epithelial compartment^10^. Stem cells have the capacity for unlimited growth and can give rise to further stem cell progeny or cells with a more limited proliferative index known as transit-amplifying cells^11^.

The hierarchical organisation of morphologically heterogeneous cell types in normal tissue was first demonstrated in seminal work by Barrandon and Green^12^. This apparent heterogeneity, in not only morphology but also functional expression patterns, is thought to echo the varying developmental and maturation stages of normal stem and progenitor cells^13^. Cells were shown to generate a range of clonal morphologies with varying proliferative capacities, which were termed holoclones (holo = entire), meroclones (mero = partial) and paraclones (para = beyond)^12^. These phenotypically plastic colonies are believed to derive from stem, early and late-stage transit-amplifying cells, respectively. This unique pattern of hierarchical colony formation has been demonstrated in multiple immortalised cell lines and has since become a surrogate assay for the identification and characterisation of normal stem cells^14^.

Many of the properties inherent in stem-cells are highly relevant to human cancer^15,16^. These observations have fostered the genesis of the ‘CSC hypothesis’, which is predicated on the basis that extensive tumour cell heterogeneity occurs as a direct result of the subversion of stem cell properties^13^. The CSC hypothesis states that a biologically distinct, rare subset of cells derived from the mutation of normal stem cells and their progenitors have the capacity to perpetuate the continued expansion of malignant cells such as in PCa^17–19^. In support of this hypothesis, stem-like cells capable of recapitulating the complexity of human tumours have been identified not only in haematological malignancies but also in a variety of solid tumour types including; breast^20^, glioma^21^, colon^22^, pancreatic^23^ and lung cancer^24^.

At present, there exists a significant degree of uncertainty concerning the study of PCa stem cells. While it has been postulated that many cancers are initiated and perpetuated by stem-like cells, the extent to which tumour aggressiveness is influenced by CSCs remains to be defined^16^. It remains to be determined whether this subpopulation arises as a result of malignant transformation of normal stem cells or whether differentiated cells gain mutations, which concomitantly result in the re-initiation of stem-like characteristics. It has been postulated that CSCs have the propensity to regenerate the prostate tumour following otherwise successful primary treatment^25^. They have also been implicated in the metastatic dissemination of cancer^13^. More recently, the theory that testosterone-independent primitive stem-like PCa cells contribute to disease recurrence and can be targeted with traditional chemotherapy, has received impetus from the publication of the CHAARTED trial^26^.

For these reasons, there is a clinical imperative to characterise this cellular population in greater detail. However, the establishment of PCa stem cell-targeting therapeutics is reliant upon the development of reliable and reproducible methods for the isolation and maintenance of CSCs of known cellular phenotype. In the present study, we sought to establish a method to cultivate putative PCa stem cells, and determine whether immortalised PCa cell lines retain the hierarchical cellular framework observed in normal epithelial cells and primary tumours, and examine if the gene expression repertoires, particularly miRNA profiles, of monoclonally-derived putative stem cells reflect their developmental origin. The mRNA-miRNA network provided by these models will aid in the identification of proteomic or miRNA therapeutic targets, thus significantly improving the survival rate of patients with PCa.

## Results

### Prostate cancer cell lines generate holoclones when plated in high-salt agar

**Figure 1:**
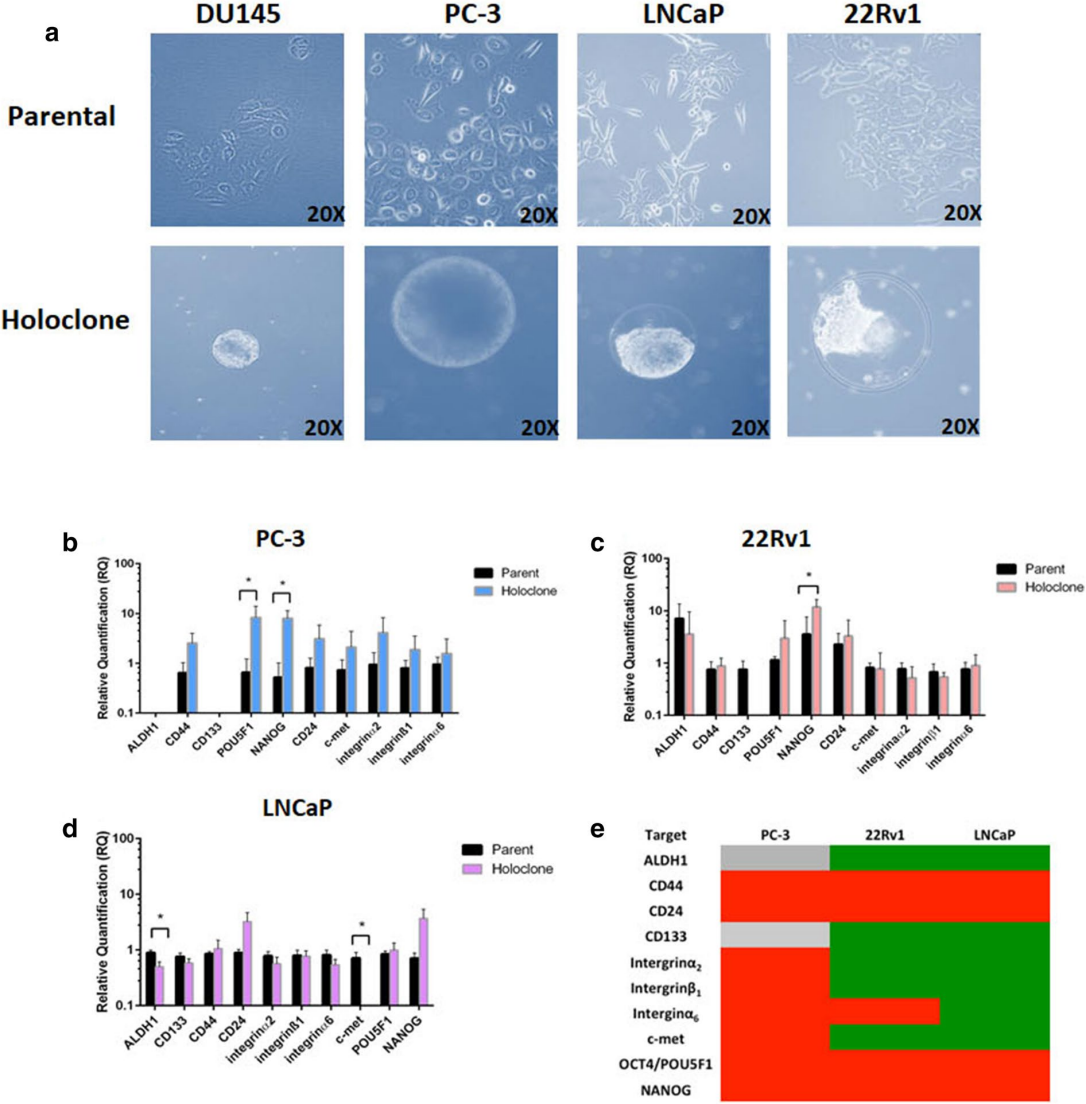
(**a**) Representative bright field images of parental cells and holoclones derived from DU145, PC-3, LNCaP and 22Rv1 prostate cancer cell lines at approximately 5–7 weeks following initial plating in high-salt agar. (**b**–**e**) Stemness gene expression profile in holoclones and parental cells derived from high-salt agar. (**b**–**d**) Relative quantification of change in expression of stem-associated genes (ALDH1, CD44, CD133, POU5F1, NANOG, CD24, c-Met, integrin α2, integrin β1 and integrin α6) in PC-3, 22Rv1 and LNCaP-derived holoclones, which were normalised to parental controls. (**e**) The associated heat map summarising changes in gene expression (holcolones versus parent) (Grey: undetected, green: downregulated, red: upregulated). Data represented as Mean ± SEM (*p < 0.05, unpaired Student’s two-tailed *t* test, n = 3).

Basal levels of stem cell markers were assessed in four prostate cancer cell lines using FACS (Supplementary Fig. 1, Supplementary Table 1). In order to enrich for cells with a putative stem cell phenotype, the panel of cell lines (DU145, PC-3, 22Rv1 and LNCaP) were cultured in high-salt agar that was optimised in our laboratory for putative CSC isolation, with the effectiveness for holcolone growth demonstrated using ovarian, melanoma and thyroid cancer cell lines (*unpublished data*). To the best of our knowledge, this is the first study to examine the use of high-salt agar to cultivate putative PCa stem cells. The 22Rv1 and LNCaP parental cells generated holoclones more efficiently than PC-3 and DU145 cells (efficiency data not shown) with at most 2–4 holoclones observed in each plate over a four week time frame. Holoclones were observed as densely arranged bundles of cells, spherical in morphology due to the presence of a defined halo structure surrounding the cells in the agarose surface (Fig. 1a). Cells developed within this halo and generally extended to its boundaries; however, they were not observed growing beyond the perimeter of this structural anomaly. The precise function of this apparent halo has yet to be elucidated; however, it does not appear to be unique to PCa cells as it has previously been observed within our laboratory for melanoma, thyroid and ovarian cancer cell lines (*unpublished data*). The low-density culture of immortalised cells in a high-salt environment has been postulated to select for robust cells with self-renewal potential^27^. The data reported here show that this technique was highly inefficient with PCa cells, as often no holoclones were observed across multiple agarose plates. In particular, DU145 showed poor holoclone formation efficiency (efficiency data not shown).

### Stemness gene signature is altered in holoclones cultured in high-salt agar

The putative stem-like phenotype of holoclones from PC-3, LNCaP and 22Rv1 cells, generated using the high-salt agar technique, was assessed through the expression levels of a panel of key stem cell-associated genes (ALDH1, CD44, CD133, POU5F1, NANOG, CD24, c-Met, integrin α2, integrin β1 and integrin α6) (Fig. 1b–e). While there was a trend towards an increase in expression of CD44 and CD24 in PC-3 and 22Rv1-derived ‘stem cells’, POU5F1 was significantly increased in 'stem cells' from PC-3 cells only (p < 0.05) (Fig. 1b). NANOG mRNA was significantly increased in PC-3 (Fig. 1b) and 22Rv1 (Fig. 1c) holoclones relative to parental cells (p < 0.05). Of note, both ALDH1 and c-Met were significantly down-regulated in LNCaP (Fig. 1d) holoclones compared with parental cells (p < 0.05). A variable expression pattern was observed amongst cell lines for the other genes (Fig. 1b–d). While CD44 and integrin α2β1 have been increased in some studies examining holoclones, our data for PC-3 is in line with a study by Zhang and Waxman^28^ which found that PC-3 holoclones did not show enriched expression of these markers. It should be noted, however, that this is the first study to exmaine stem markers in holoclones cultured from high-salt agar, and thus differing expression patterns is likely reflective of the methodology used. A summary of these data (holoclones versus parental cells) are represented as a heatmap in Fig. 1e.

### Monoclonal cultivation of PC-3 and DU145 cells generate morphologically heterogeneous colonies

**Figure 2:**
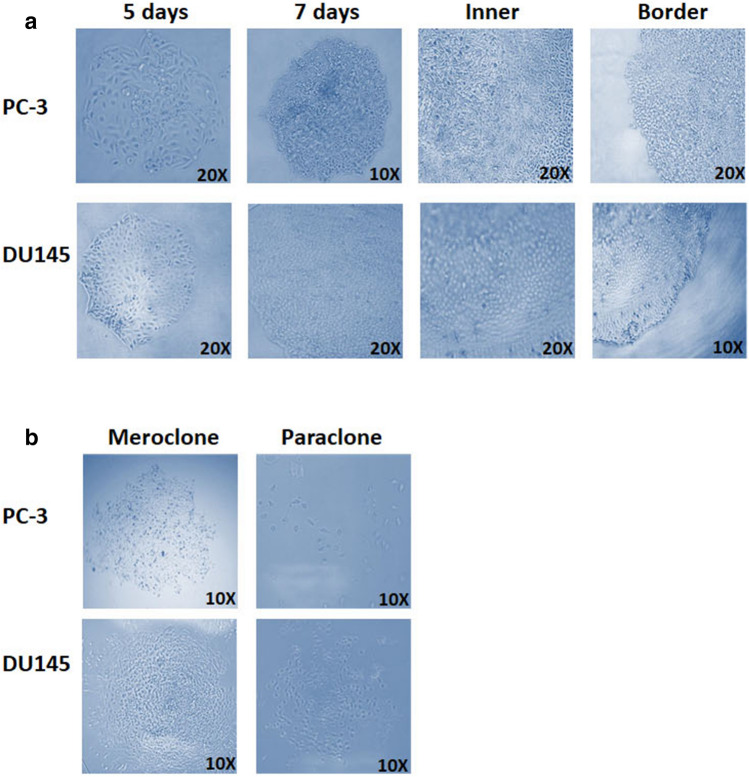
PC-3 and DU145 generate morphologically heterogeneous colonies using a colony formation assay. (**a**) Representative images of holoclones 5 days and 7 days following plating. Also shown are the inner cellular composition of holoclones and the border composition. (**b**) Representative images of meroclones and paraclones. (Images are representative of three independent experiments).

We assessed the colony formation assay or ‘monoclonal cultivation’ assay as an alternative method for the isolation of putative CSCs. 22Rv1 and LNCaP cells were unable to form colonies using monoclonal cultivation (data not shown). When plated as single-cells, PC-3 and DU145 displayed different abilities to generate phenotypically plastic colonies (Supplemental Fig. 2), with PC-3 demonstrating the ability to generate more Colony Forming Units compared to DU145 for all types of clones formed (Supplemental Fig. 2). Visually the morphologies of these colonies closely resembled those produced by stem and late-amplifying cells of the normal prostate epithelium (Fig. 2a). As previously mentioned, clones were either classified as holoclones, meroclones or paraclones based on morphological features. Holoclones were comprised of small, tightly packed cells and possessed smooth, defined colony borders (Fig. 2a). Paraclones contained dispersed larger cells, while meroclones exhibited an intermediate morphology containing a dichotomy of cell shapes and sizes (Fig. 2b). Their borders were often more fragmented than those observed in holoclones. Additional assays demonstrated that only holoclones, not meroclones or paraclones, had the ability to regenerate all colony morphologies (Supplemental Fig. 3).

### Holoclones-derived by monoclonal cultivation are enriched for stem-associated markers

**Figure 3:**
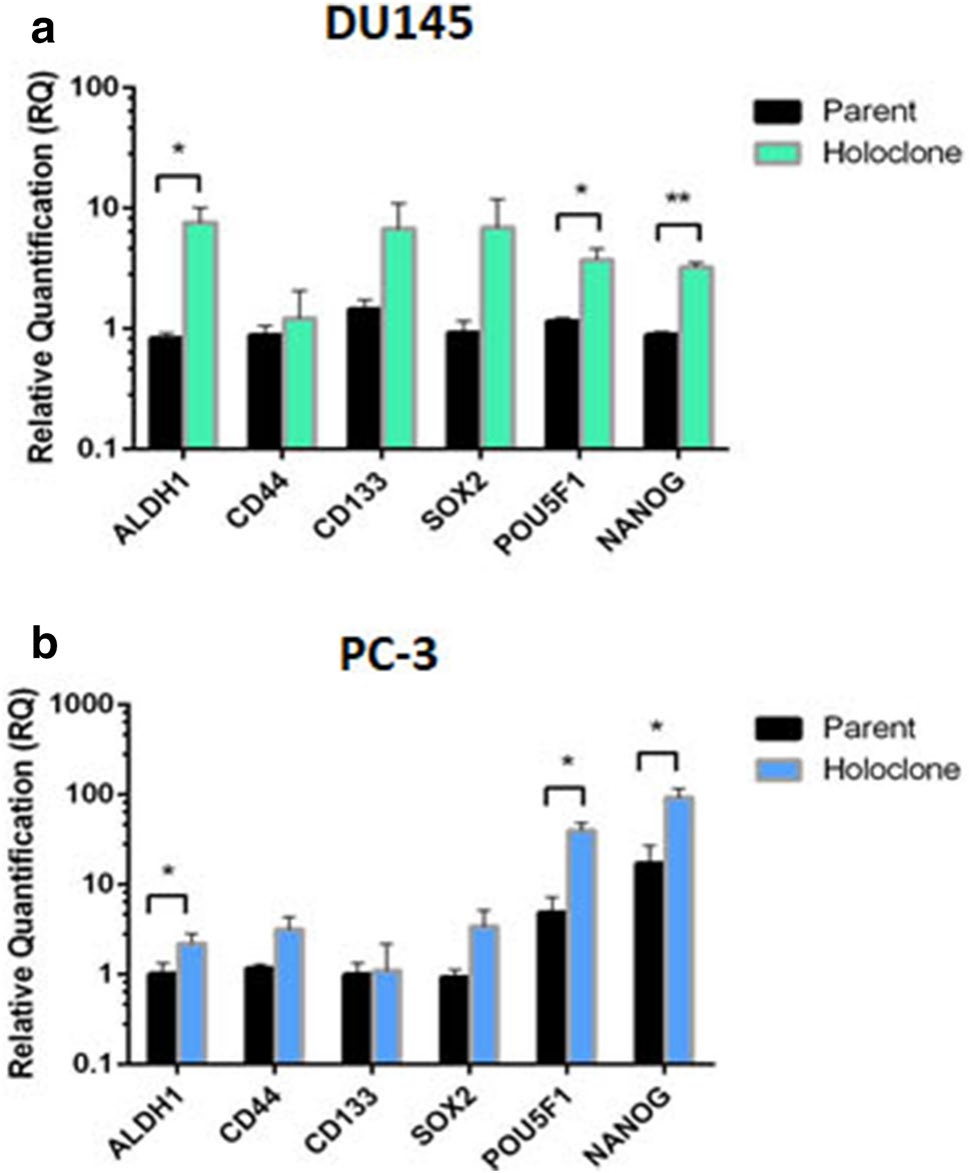
Expression of stem cell-associated markers in holoclones-derived from the colony forming assay in (**a**) DU145 and (**b**) PC-3 cells. Relative quantification of change in expression of stem-associated genes in holoclones normalised to parental controls. Data represented as Mean ± SEM. (*p < 0.05, **p < 0.01, unpaired Student’s two-tailed *t* test, n = 3).

**Figure 4:**
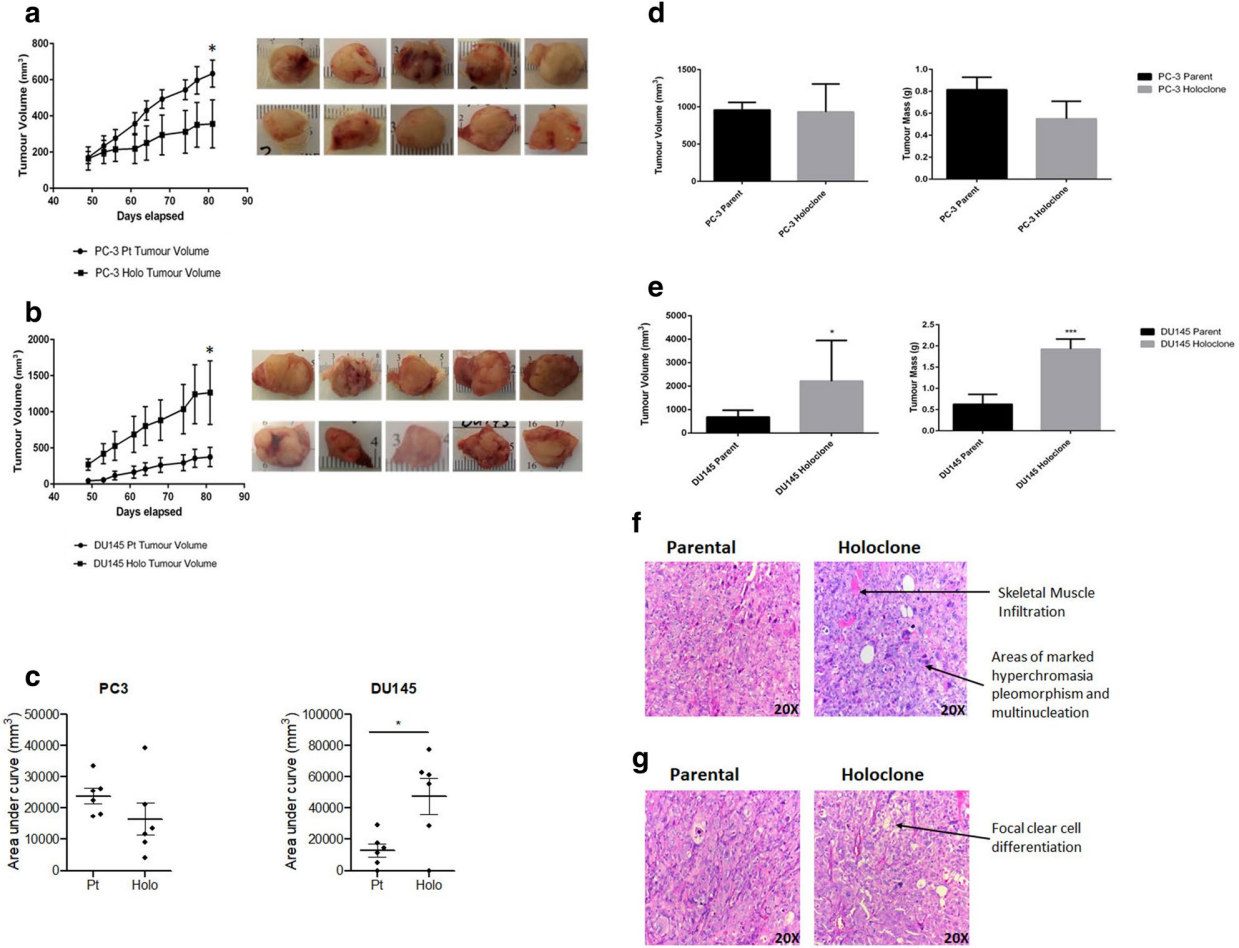
Xenotransplantation study of PC-3 and DU145 parental and holoclone-derived tumours. Growth curves for PC-3 (**a**) and DU145 (**b**), following injection of 3,000 parental and holoclone cells into NOD/SCID mice. PC-3 parental tumours were larger than holoclone tumours (day 81 post injection; p < 0.05), however holoclone-derived tumours were larger than parental cells in DU145 at day 81 post injection (p < 0.05). Data represented as Mean ± SEM (*p < 0.05, ANOVA using Tukey’s post hoc test, n = 6). The area under curve (AUC) for DU145 and PC-3 tumour growth for parent cells (Pt) and holoclones (Holo) at each time-point was estimated and represented as a scatter plot (**c**). Differences in AUC between groups were calculated using a two-tailed unpaired Student's *t* test. Data are represented as the Mean ± SEM (*p < 0.05; AUC tumour volume DU145 Pt cells *vs*. Holo, n = 6). Ex-vivo tumour volume and tumour mass in PC-3 (**d**) and DU145 (**e**) parent and holoclone-derived tumours. Data represented as Mean ± SEM (*p < 0.05, ***p < 0.001, unpaired Student’s two-tailed *t* test, n = 6). (**f**) Representative H&E-stained tumour sections from PC-3 parental cells and holoclones. The parental tumour was found to infiltrate the skin and extensive necrosis was present. In the holoclone-derived tumours, cells were markedly pleiomorphic with very prominent nucleoli. There was abundant mitosis, widespread vascular invasion and focal necrosis present. Tumour was also found to infiltrate the skeletal muscle. Arrows depict areas of interest. (**g**) Representative DU145 parent and holoclone-derived H&E stained tumour sections. In parental cells, muscle was infiltrated by very poorly differentiated carcinoma with marked pleiomorphism. Tumour was comprised of mainly large cells with morphologies consistent with polylobated and prominent nucleoli, or multi-nucleated. Very apparent apoptosis and mitosis were also noted. However, focal areas of clear cell change were identified, and muscle infiltration was found to be much more widespread within holoclone-derived tumours. Arrows depict areas of interest.

The expression of several key stem cell-associated markers (ALDH1, CD44, CD133, SOX2, POU5F1 and NANOG) were analysed in both DU145 and PC-3 holoclones. All markers were increased in holoclones compared with parental cells (Fig. 3). Expression levels of ALDH1 (p = 0.04), NANOG (p = 0.001) and POU5F1 (p = 0.04) were significantly higher in DU145 holoclones than in parental cells (Fig. 4a). Similarly, ALDH1 (p = 0.03), POU5F1 (p = 0.02) and NANOG (p = 0.04) were significantly higher in PC-3 holoclones relative to their parental cells (Fig. 3b). Interestingly, when cultured in stem cell media, the mRNA levels of SOX2, POU5F1 and NANOG, were shown to be higher in DU145 prostatospheres^29^ compared with parental cells (p < 0.05) (Supplemental Fig. 4). The stem cell-associated markers were not assessed in the meroclone or paraclone population as previously published data indicates that these populations do not display stemness features^30^. In our study, we have also demonstrated that it was only holoclones which had the capability to generate all colony morphologies; meroclones could generate meroclones and paraclones; paraclones could generate only paraclones (Supplemental Fig. 3).

### Parental cells and holoclones form tumours in NOD/SCID mice

Although clones generated from both high-salt agar and the colony formation assay displayed stem-like signatures, the capacity to regenerate tumour pathophysiology upon xenotransplantation is considered an essential benchmark in defining CSCs^16^. To assess this in vivo, 3,000 holoclone (derived from the monoclonal cultivation assay) and parental cells from DU145 and PC-3 were injected subcutaneously into the flanks of male NOD/SCID mice. Approximately 49 days post injection, palpable tumours were evident for both parental cells and holoclones. Mice were sacrificed on day 87, with no evidence of metastases upon dissection. Tumour growth curves for parent and holoclone cells are shown for PC-3 (Fig. 4a) and DU145 cell lines (Fig. 4b). PC-3 parental tumours were significantly larger than tumours derived from holoclones (p < 0.05). Of interest however, the opposite was observed for DU145 cells (p < 0.05). AUC for tumour growth curves derived from DU145 and PC-3 parent cells and holoclones were calculated at each time-point (Fig. 4c). While there was no significant difference in PC-3 cells *vs*. holoclones (p = 0.127), there was a statistically significant increase in AUC between DU145 parent cells versus holoclones (p = 0.020). There were no differences in tumour volume or mass ex vivo between PC-3 parental cells and holoclone-derived cells (Fig. 4d). However, animals injected with DU145 holoclone cells generated tumours, which were significantly larger than parental tumours both in terms of volume (p < 0.05) and tumour mass (p < 0.001) (Fig. 4e). Histological analysis of tumours using Haematoxylin & Eosin (H&E) revealed similar histological features between parental and holoclone-derived cells. In PC-3 cells (Fig. 4f), extensive necrosis was evident across tumours. Holoclone-derived tumours however were markedly pleomorphic, with highly prominent nucleoli and abundant mitoses (Fig. 4f). Skeletal muscle infiltration was also evident in tumours derived from PC-3 holoclones (Fig. 4f). Focal areas of clear cell change were observed in the DU145 holoclones (Fig. 4g).

### E-cadherin expression is altered between parental and holoclone tumours in vivo

**Figure 5:**
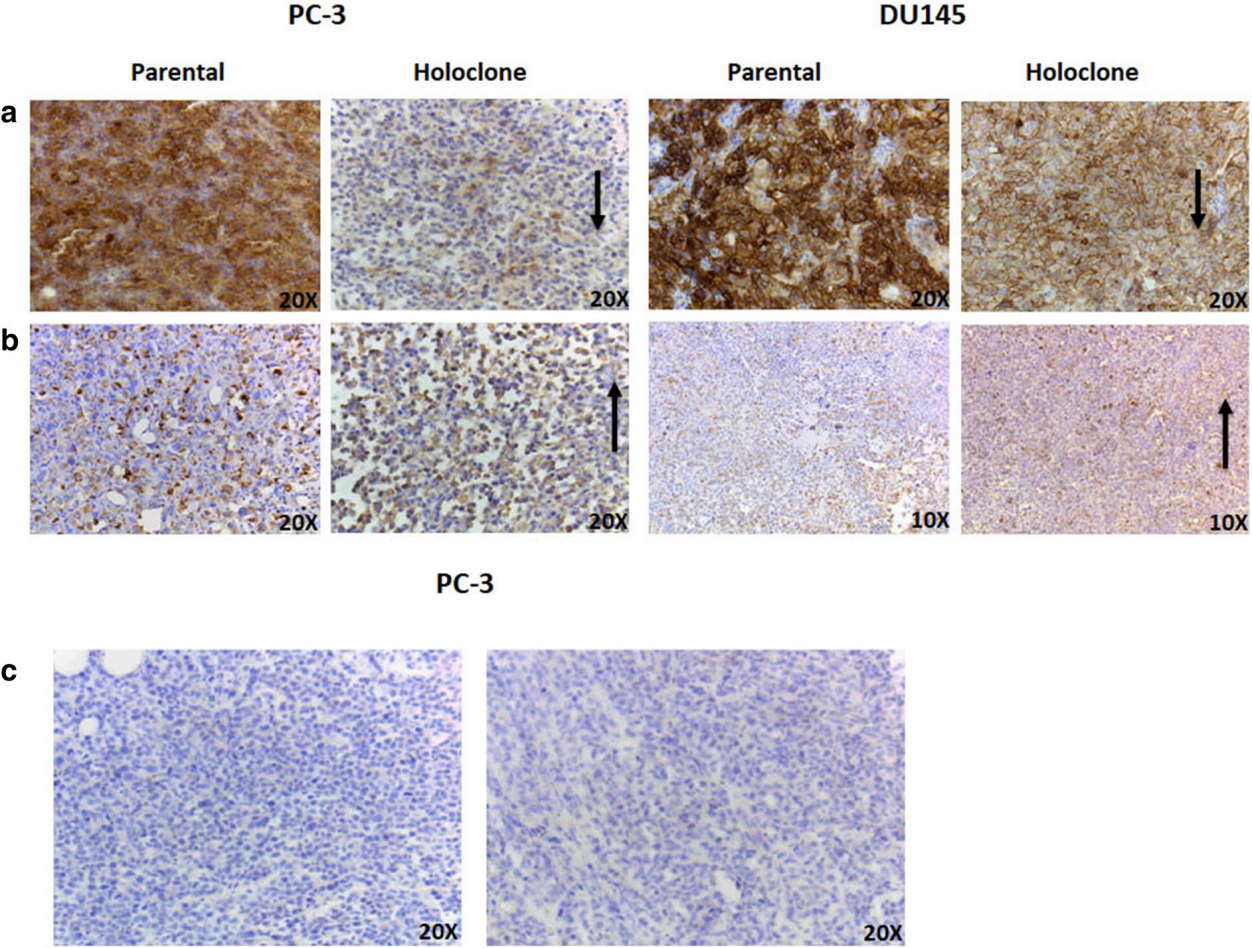
Representative E-Cadherin, Vimentin and CD34 staining in parent and holoclone-derived tumour sections. (**a**) E-cadherin expression in PC-3 and DU145 parental and holoclone tumours. E-cadherin expression was observed as diminished in the holoclone-derived tumour sections. Arrows depict areas of interest. (**b**) Vimentin expression in PC-3 and DU145 parental and holoclone tumours. Little difference in vimentin expression was observed between parent and holoclone samples. Arrows depict areas of interest. (**c**) CD34 staining in PC-3 parental and holoclone-derived tumour. No detectable vessel staining was observed in either parental or holoclone tumour samples. Representative images from three tumour samples.

In the absence of evident metastases upon dissection, we sought to investigate whether holoclone-derived tumours preferentially exhibit a loss of epithelial morphology and a concomitant acquisition of a mesenchymal phenotype. Immunohistochemistry (IHC) was employed to examine the expression of E-cadherin and vimentin in a subset of representative parent and holoclone-derived (n = 3) tumour sections (Fig. 5). Qualitatively, a decrease in E-cadherin expression was observed in both DU145 and PC-3 holoclone-derived tumours when compared to parental tumours (Fig. 5a). However, no discernible difference in vimentin expression was observed between parent and holoclone tumour pairs in either cell line (Fig. 5b). CSCs have previously been implicated in the development of angiogenesis. Thus, to determine whether a differential degree of vasculature was present between parental and holoclone-derived tumours, a subset of tumour sections (n = 3) were chosen for IHC staining with the vascular marker, CD34. No positive staining for CD34 (Fig. 5c) was observed in PC-3 or DU145 tumour samples (parental and holoclone-derived) (data not shown for DU145). Although not formally examined, there was no evidence of neuroendocrine de-differentiation in the tissues sampled.

### NGS analysis demonstrated modified global gene expression between parental cells and holoclones in vitro and in vivo

**Figure 6:**
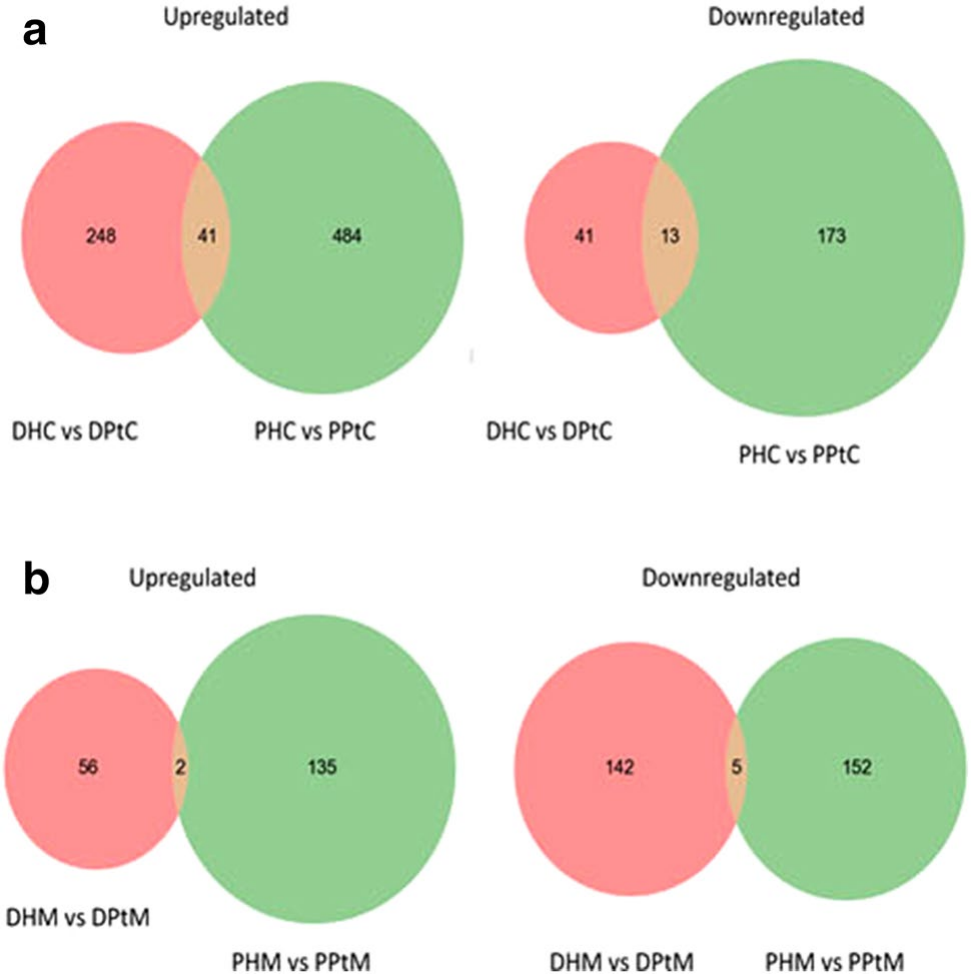
Venn diagrams detailing shared and distinct gene expression among DU145 and PC-3 parent and holoclone cells (**a**) and murine-derived (**b**) samples. (**a**) Forty-one genes were found to be commonly upregulated in holoclones (when compared to their parental counterparts derived from DU145 and PC-3 cells). Thirteen downregulated genes were shared by PC-3 and DU145-derived holoclones (n = 1). *DHC* DU145 holoclone cells, *PHC* PC-3 holoclone cells, *DPtC* DU145 parental cells, *PPtC* PC-3 parental cells. (**b**) Two genes were found to be commonly upregulated in PC-3 and DU145 holoclone-derived tumours (when compared to those generated by respective parental cells). 5 genes were commonly downregulated in both PC-3 and DU145 holoclone-derived tumours (n = 1). *DHM* DU145 holoclone murine tumour, *PHM* PC-3 holoclone murine tumour, *DPtM* DU145 parental murine tumour, *PPtM* PC-3 parental murine tumour.

NGS analysis was performed to identify the myraid of molecular alterations in our holoclone population, thus aiding in a deeper understanding of the regulatory mechanisms, which underpin functional plasticity within this cellular population. Forty-one genes were identified as upregulated in holoclones compared to parental cells (of both cell lines), while 11 genes were identified as downregulated in holoclones (Fig. 6a; Supplementary Table S2). In murine samples, two genes were found to be upregulated in holoclone tumours compared to parental cell-derived tumours. In addition, two genes were found to be downregulated in holoclone-derived tumours (Fig. 6b, Supplementary Table S3). Gene ontology software analysis, using DAVID, of PC-3 and DU145 holoclones identified alterations in pathways involved in organ development, haematopoiesis, regulation of apoptosis, regulation of cell communication, response to stress and anatomical structure development (data not shown). Notably, genes highly upregulated in holoclones included EGR1, KITLG, NR4A2 and TXNIP, all of which have well-documented associations with haematopoiesis.

### NGS analysis identified altered miRNA expression in parental cells and holoclones in vitro and in vivo

**Figure 7:**
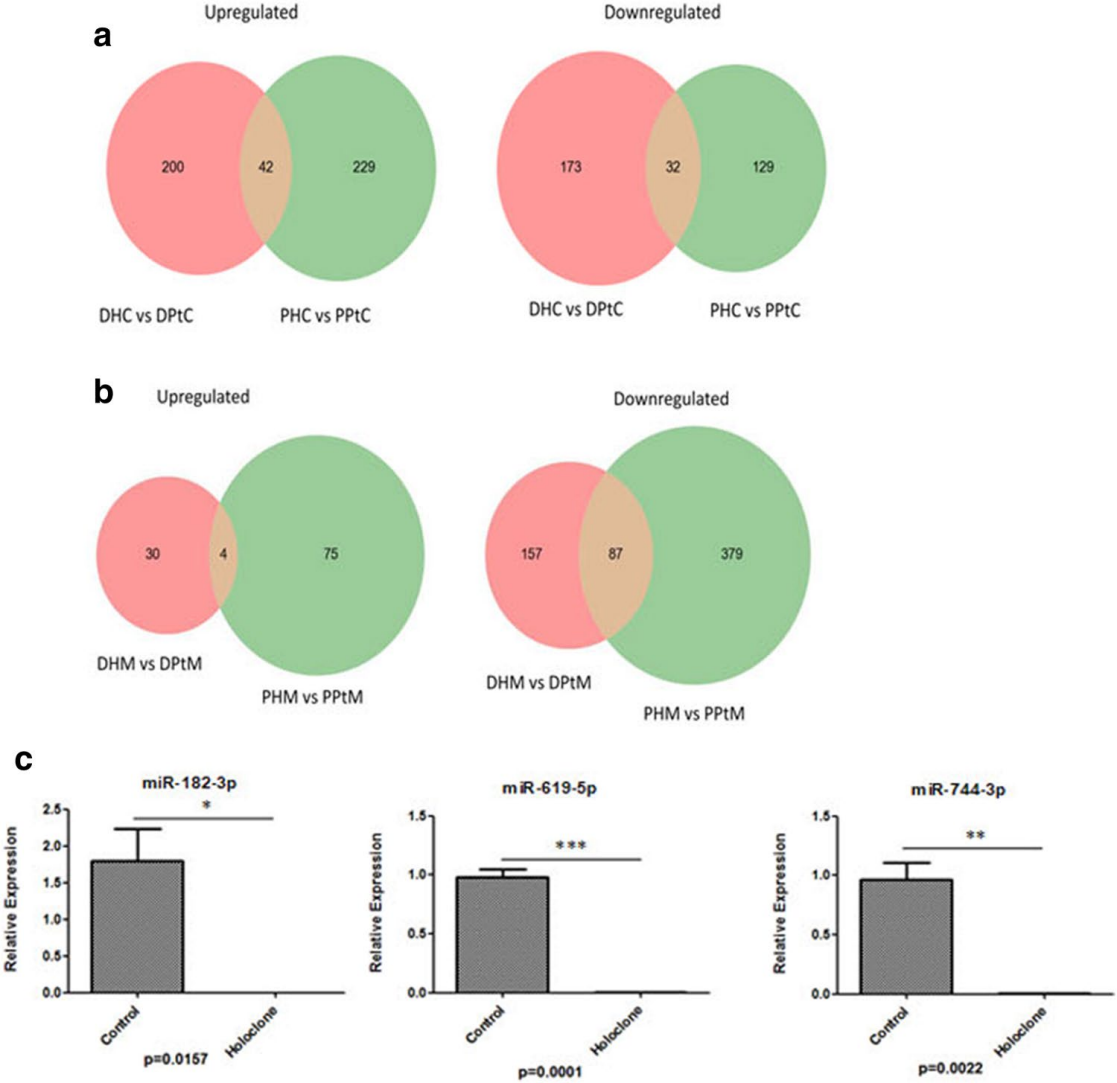
Venn diagrams detailing shared and distinct miRNA expression among DU145 and PC-3 parent and holoclone in cellular (**a**) and murine (**b**) samples. (**a**) Forty-two miRNAs were identified as upregulated in holoclones of both cell lines, while 32 miRNAs were commonly downregulated. (n = 1) *DHC* DU145 holoclone cells, *PHC* PC-3 holoclone cells, *DPtC* DU145 parental cells, *PPtC* PC-3 parental cells. (**b**) Only 4 miRNAs were found to be commonly upregulated by holoclone-derived tumours, while a much larger set of 87 miRNAs were found to be downregulated in holoclone-derived tumours. (n = 1) *DHM* DU145 holoclone murine tumour, *PHM* PC-3 holoclone murine tumour, *DPtM* DU145 parental murine tumour, *PPtM* PC-3 parental murine tumour. (**c**) Validation of miR-182-3p, miR-619-5p and miR-744-3p in the PC-3 cellular-derived holoclones. Data is represented as Mean ± SEM (*p < 0.05, **p < 0.01, ***p < 0.001, unpaired Student’s two-tailed *t* test, n = 3).

Following global gene expression analysis, miRNAs were also analysed in an attempt to identify unique miRNA signatures, which may act as important modifiers of PCa stem cell properties. In vitro, 42 miRNAs were identified as upregulated in holoclones derived from PC-3 and DU145 cells relative to parental cells, while 32 miRNAs were commonly downregulated (Fig. 7a; Supplementary Table S4). Eight miRNAs were selected for validation (miR-20b-5p, miR-10b-5p, miR-619-5p, miR-744-3p, miR-4706, miR-500a-3p, miR-182-3p and miR-340-5p) in PC-3 and DU145 cells. These were selected based on their importance in PCa stemness. Of these miRNAs, 7 demonstrated a similar trend in expression; however the change in miRNA expression did not reach significance between holoclones and their parental cell counterparts. However, in PC-3 holoclones, miR-182-3p (p < 0.05), miR-619-5p (p < 0.001) and miR-744-3p (p < 0.01) (Fig. 7c) were significantly decreased relative to parent cells. Additional validations are provided in Supplemental Fig. 5. In the murine samples, only 4 miRNAs were found to be commonly upregulated in holoclone-derived tumours (hsa-miR-376b-5p, hsa-miR-628-5p, hsa-miR-136-5p and hsa-miR-4687-5p) (Venn diagram provided in Fig. 7b), while a much larger set of 87 miRNAs were found to be downregulated in holoclone-derived tumours (Fig. 7b; Supplementary Table S5).

### Network of miRNA: Target interactions (MTIs)

Genes and miRNAs identified as being dysregulated were further investigated to identify interactions using databases of miRNA: Target Interactions (MTIs). The results from each separate database were concatenated and visualised as directed acyclic graphs (Supplemental Fig. 6a–c). This analysis allowed for the visualisation of potentially criticial regulatory networks, which are dysregulated in PCa holoclones. Full MTI network tables can be found in Supplementary Tables S6 and S7. A definitive ‘anti-correlation’ was identified between interacting miRNA-gene pairs, which theoretically confirmed the efficacy of the sequencing run. For example, miR-27a, which was identified as downregulated in holoclones, was found to target KITLG and as previously mentioned this gene was identified as highly upregulated in holoclones (Supplementary Fig. S6a). This analysis allows the visualisation of potentially critical regulatory networks, which are dysregulated in PCa holoclones.

## Discussion

The ontogeny of PCa stem cells is poorly understood and it remains to be determined whether this subpopulation arises as a result of malignant transformation of normal stem cells or whether differentiated cells gain mutations, which concomitantly result in a re-initiation of stem characteristics. It has been postulated that CSCs have the propensity to regenerate a tumour following otherwise successful primary treatment. They have also been implicated in the metastatic dissemination of cancer^13^. For these reasons, there is a clinical requisite to characterise this cellular population in greater detail and determine its therapeutic importance. Devising new methodologies to enrich for stem-like cells for potential purification and characterisation of their biological properties are warranted in PCa.

A number of studies in prostate^30–33^, pancreatic^34^, colorectal^35^, breast^36^ and head & neck^37^ cancer cell lines have reported the use of colony morphology as a surrogate marker in defining stem cell-derived colonies, transit-amplifying cells and differentiating cells. Despite the disparity between these studies, most if not all, previous studies conclude that holoclones have a greater ability to be passaged in bulk culture^30,34^ or by serial cloning^31,32,34,38^, than paraclones, which tend to have a very limited proliferative potential. Data from these studies demonstrate that meroclones could not be cultivated^31,32^ compared to holoclones, which could be propagated for more than 6 months^30^. The ability of cells derived from meroclones to generate secondary holoclones was observed in one study, but few holoclones were formed from meroclones^34^. While the gold standard for deducing CSC activity of specific cell subpopulations is their ability to form tumours and initiate serially transplantable tumour development^39,40^, PCa is a very heterogeneous tumour in which the CSC pool contains heterogeneous tumorigenic subsets that possess distinct tumour-initiating properties^41^.

In this study, two in vitro models were used to evaluate the efficiency of isolating PCa-derived holoclones enriched for stem cell properties, using high-salt agar growth and monoclonal cultivation. When cultivated in high-salt agar, cell lines displayed differential formation efficiency. Multiple cell types in the prostate gland have been considered as potential cell(s) of origin for tumour development^42^, with cellular differentiation states impacting cell lineages and the origin of prostate cancer^43^. The cell lines in this study were from different sites^44–47^ and display differential metastatic potential^48^. Thus, it is reasonable that differential formation efficiency may, in part, be associated with the origin of the cell lines. While there have been few studies examining high-salt agar as a means of cultivating putative CSCs in cancer^49,50^, to our knowledge this is one of the first studies to examine this model in the context of PCa. Holoclones express high levels of stem and progenitor cell markers such as CD133, CD44, and α2β1 integrin. In order to initially characterise stem cell–associated properties in four PCa cell lines (DU145, PC-3, 22Rv1, LNCaP), all cell lines were immunophenotyped for the expression of these markers. Previous studies have shown that CD44+ and CD44+/integrin α2β1+ PCa cell populations are enriched in tumorigenic and metastatic CSCs, whereas integrin α2β1+ PCa cells most likely mark fast-proliferating tumour progenitors^51^. Our data show that stem cell characteristics (CD44+/integrin α2β1hi/CD133+) are maintained by a small population of epithelial cells cultured long term ranging from 2.05% in DU145 cells to 16.9% in LNCaP cells (co-expressing all three markers). It has been widely reported that only a small fraction of cells (approximately 0.1%) within a tumour exhibit stem-like characteristics such as indefinite self-renewal, differentiation and the ability to undergo asymmetric division^52^. Based on the expression profile demonstrated in our panel of PCa cell lines, the presence of a larger stem fraction in immortalised PCa cell lines is indicated. This may be due to several factors. Firstly, the biological nature of cancer cells may be altered in vitro through multiple passaging, perhaps creating a bias for CD133+ cells, which may concomitantly increase the stem-like population.

Furthermore, previous attempts to isolate and culture highly tumorigenic CD133+ cells from the LNCaP cell line, have demonstrated that despite being cultured from a > 98% pure population of CD133+ cells, only 6.15% of CD133+ cells remained following two weeks of culture, indicating that in vitro expansion of this population will result in differentiation and a consequent loss of the stem phenotype. This was also observed in a study by Wei et al.^53^, in which a CD44+/integrin α2β1hi/CD133+ population was isolated from DU145 cells. Upon culturing this population in medium containing 10% FBS, the majority of cells differentiated as corroborated by an increase in cytokeratin 18 (CK18) expression. Overall, while the limitations associated with the isolation and subsequent expansion of a CD44+/integrin α2β1hi/CD133+ population would appear to preclude its successful application to the large-scale analysis of prostate CSCs in vitro, it does provide an insight into the proportion of cells within established cell lines, which potentially retain stem characteristics despite long-term culture.

In high-salt soft agar assays, PCa cell-derived holoclones were formed, albeit at differential efficiencies. Gene expression levels for enriched stem cell-associated markers within these holoclones identified variable expression across all cell lines, most notably in PC-3-derived holoclones and those from 22Rv1 cells, showing significantly increased expression of the CSC markers, Nanog (PC-3, 22Rv1) and POU5F1 (PC-3). This variability in expression may suggest that this high-salt technique may not enrich for a pure stem cell population, thus holoclones generated in this manner may represent a mixed population of cells including stem and late progenitor cells. The ambiguity surrounding the ontogeny of these resulting holoclones coupled with the relative inefficiency of this technique indicate that this may not be the most effective model for the isolation and characterisation of PCa stem cells, this is in contrast to other studies^49^. A similar method reported by Olszewski et al. reported the culture and preservation of human epidermal stem cells in anhydric sodium chloride (0.9%) for several weeks following transplantation giving rise to keratinocyte progenies^27^.

The extension of the colony forming assay to immortalised cancer cell lines has more recently identified putative CSCs in normal tissues such as human hair follicles^54^, ocular surface^55^, human epidermal keratinocytes^56^ and in various cancers such as prostate^30^, glioma^57^ and pancreatic^34^. This technique generated phenotypically plastic colonies with hierarchical proliferative abilities consistent with that of stem, transit-amplifying and differentiated cells. While 22Rv1 and LNCaP cells failed to form colonies, PC-3 and DU145 cells successfully generated morphologically heterogeneous holoclones, meroclones and paraclones. These colonies were identified based upon their differential morphological features and closely resembled those described in previous studies^34^. Examination of colony composition demonstrated that total colony formation by PC-3 cells was greater than those formed from DU145 cells. Of these, approximately 1.85% of DU145 cells and 3.05% of PC-3 cells could generate holoclones. These findings are in keeping with previous reports, which indicate that the stem cell niche can comprise approximately 1–10% of the malignant population^28,58^. These findings are supported by other studies, which allude to the hypothesis that the major difference between holoclones and meroclones derived from a cancer cell line, is the proportion of stem cells within each colony and not the presence or absence of stem cells^59^. These findings may reflect the properties of cancer as opposed to normal cells, perhaps indicating that the hierarchy of stem cells is more extensive in cancer.

Furthermore, second-phase plating demonstrated that dissociated holoclones generate a higher number of secondary holoclones compared to naïve parental cells, highlighting the powerful proliferative capacity of these holoclones. We also found that only holoclones possessed the ability to regenerate all colony morphologies on subsequent resubmission to monoclonal cultivation. However, a study by Beaver and colleagues has demonstrated that meroclones from DU145 could produce hololcones^59^; however meroclones were found to regenerate only meroclones and paraclones in our study. The stemness phenotype of clonally cultivated holoclones was assessed by examining the expression of stem cell-associated genes. Both PC-3 and DU145 holoclones exhibited a significant overexpression of NANOG, POU5F1 and ALDH1 mRNA. The relative expression ALDH1 was greater in holoclones derived from DU145 prostate cells than in those derived from PC-3 cells. While CD44, CD133 and SOX2 were not significantly altered, there was a trend towards an increase in expression in holoclones of both PC-3 and DU145 cell lines relative to their parental cells. Transcription factors, which play a critical role in reprogramming cells to pluripotency have also been identified in human cancers, including PCa and include POU5F1, SOX2 and ALDH1^60^. ALDH activity is a marker of CSCs and is involved in oxidation of aldehydes in normal stem cell function. Its expression is essential for stem cell survival and early differentiation and has been shown to be a marker of normal haematopoietic, mammary and neural stem cells^61–63^. In a study by Doherty et al.^64^, a subpopulation of high expressing ALDH PC-3 cells was isolated, which were highly proliferative and clonogenic in comparison to low-expressing ALDH and unsorted cells. The ALDH high-expressing cells gave rise to significantly higher numbers of holoclones in comparison to ALDH low-expressing cells, suggesting that ALDH expression enriches for a more primitive cell type. The authors noted that while ALDH expression enriches for holoclones, these are largely negative for ALDH expression. Within the high-expressing ALDH population, some cells have the capacity to give rise to holoclones, however once in culture; the expression of ALDH may be switched off in the progeny of these cells.

The capacity to regenerate tumour pathophysiology upon xenotransplantation is now considered an essential benchmark in defining CSCs. Previous studies have shown that only holoclones are tumorigenic in vivo^30^ and that holoclones can form larger, faster growing tumours than paraclones^35^. Using a xenograft model with PC-3 and DU145, on day 88 post injection there was a significant difference in tumour volume observed, with PC-3 parental tumours larger than holoclone-derived tumours, while DU145-derived holoclone tumours were larger than corresponding parental cells. Tumour volume was also examined using the AUC method (provides a single integrated value that reflects the entire tumour growth curve and allows easier comparisons between groups), which demonstrated significantly larger tumour volumes in DU145 holoclones compared to parental cells. This reflects ex-vivo tumour volume and mass, which were only significantly different in the DU145 cell line. In particular, DU145 holoclones generated larger, more invasive tumours than their differentiated parental cells indicating an altered behavioural pattern for prostate holoclones. The morphological features of tumours derived from these holoclones closely resembled those of parental cell tumours upon examination by H&E. This apparent divergence in these in vivo growth characteristics observed between PC-3 and DU145 holoclones may suggest phenotypic and functional heterogeneity present within the holoclone fractions. In order to explore this concept further, it will be necessary to elucidate the relationship which exists between the CSC population as a whole and their differentiated counterparts. As previously suggested, the behaviour of CSCs may be profoundly altered when they are removed from the bulk, differentiated population^65^. However, a detailed genetic analysis will be paramount to elucidating the complex mechanisms, which govern this ‘moving dynamic target’ cellular population.

While our in vivo study yielded interesting results, we acknowledge that this study has a number of limitations. We did not perform serial tumour transplantations of our holoclones, which tends to be the ‘gold standard’ methodology for the identification of CSCs. Thus, we were limited to the assessment of inherent CSC properties from holoclones and the cell lines from which they were derived. The contrasting in vivo results between PC-3 and DU145 may be reflective of the possible inherent phenotypic and functional heterogeneity within the holoclone fractions from both cell lines. Consequently, it is possible that serial transplantation of both parental and holoclone-derived cells may have produced holoclone-derived tumours with enhanced CSC activity, and therefore enhanced differences between holoclone and parental-derived tumours. Additionally, in vivo studies with meroclones and paraclones were not part of this study. The literature is conflicting regarding the stem-like capabilities of these clones, with most concentrating on holoclones as those which have the capacity to initiate tumours in vivo^30,66^*.* In our study, during in vitro monoclonal cultivation of prostate cancer cell lines—holoclones, meroclones and paraclones derived thereof, showed that while holoclones could be maintained during this process, meroclones and paraclones were unable to grow under these experimental conditions, thereby indicating that meroclones and paraclones do not possess self-renewal ability typical of stem-like cells. This is in line with a study by Zhou et al.^66^. In contrast, a study in DU145 showed that holoclones and meroclones (but not paraclones) formed tumours in nude mice^59^. Meroclones had a reduced take rate and a longer latency compared with holoclones. The in vivo growth of meroclones, may be reflective of the methodology used to isolate them (serial cloning and passaging; sphere formation assay)^59^. A study involving PC-3 cells, showed some tumorigenicity for paraclones^28^ in SCID mice. However, PC-3 tumour incidence for paraclones were lower compared to holoclones, and paraclone tumours declined in size at later time points, with the study authors suggesting this indicated an inability of paraclones to sustain tumour growth. Overall, while studies are in agreement with regards the superior tumour initiator capabilities of holoclones, the data is less clear regarding meroclones and paraclones. The methodology used to isolate clones and the animal model used for in vivo studies are likely to have a significant impact on CSC studies. Therefore, serial transplantation and in vivo studies with meroclones and paraclones, were outside of the scope of our study.

While some aspects of this study were similar to others in the literature, in terms of a common theme of miRNA regulation across multiple prostate cancer cell lines and CSC populations, we report differences in miRNA and mRNA profiles^67–73^, with scope for further studies based on these data and the MTI mapping. In this study, NGS analysis identified a putative signature of PCa holoclones, which depicted a pro-metastatic and pro-angiogenic phenotype mediated by the expression of multiple genes (e.g. SCF, EGR1, BCL6, IL-24 and LPCAT1), and miRNAs indicative of a pro-metastatic and invasive profile. In addition, we have identified a complex regulatory circuitry instigating these expression patterns through computational MTI analysis. The varied expression signatures in this study and in the literature underscore the need for larger studies in prostate tissue samples using standardised methods and markers. In our study, miRNA profiling of monoclonal cultivated holoclones and parental cell counterparts identified altered expression of miRNAs, eight of which were selected for further validation based on their previously identified role in cancer stemness in PCa. Of this panel, miR-182-3p, miR-619-5p and miR-744-3p were found to be downregulated in holoclones relative to parental PCa cell lines. Recent evidence indicates that miR-182 together with Wnt/β-catenin, function as tumour oncogenes in the progression of a variety of tumours. How miR-182 regulates β-catenin signalling in PCa was reported in a study by Wang et al.^74^, whereby miR-182 activates the Wnt/β-catenin pathway by targeting multiple negative regulators of Wnt/β-catenin signalling such as GSK-3β, APC, CK1 and Axin. The authors reported that miR-182 acts as an oncogenic factor in the progression of PCa by aberrant activation of Wnt/β-catenin signalling. Other studies have suggested a biphasic role for miR-182 that may be exploited for prognostic and/or therapeutic purposes^75^. Evidence for a role of miR-744 in PCa progression has also been reported in studies where similar to miR-182, miR-744 was shown to activate the Wnt/β-catenin pathway by targeting multiple negative regulators of Wnt/β-catenin signalling, including SFRP1, GSK3β, TLE3 and NKD1. At the molecular level, NKD1 was found to be a major functional target of miR-744^76^. In a more recent study by Zhang et al.^77^, miR-744 was shown to be upregulated in PCa tissue when compared to adjacent normal tissue. Silencing of miR-744 resulted in the inhibition of cell growth and increased apoptosis. This knockdown of miR-744 resulted in activation of the adenosine monophosphate-activated protein kinase (AMPK) signalling pathway, and to a lesser extent, mammalian target of rapamycin (mTOR) signalling. These data provide evidence for a critical role of miR-744 in PCa. Using DIANA mirPath software tools^78^, an online software suite (https://www.microrna.gr/miRPathv3) dedicated to the assessment of miRNA regulatory roles and the identification of controlled pathways, KEGG pathway analysis of miR-182, miR-619 and miR-744 identified significant alteration of the miRNAs in key pathways involving fatty acid biosynthesis and metabolism, steroid biosynthesis, RNA transport and spliceosome involvement. Although not validated in this study, the changes in the expression of other miRNAs such as miR-301 and let-7b have been determined previously^79^.

Overall, our findings demonstrated the significant, reproducible upregulation of a number of genes particularly the stem cell markers NANOG, OCT4/POU5F1 and ALDH1 in prostate cancer holoclones. It was expected that the application of RNA-seq would reproduce these findings; however, the expression levels of these genes were not found to be co-ordinately upregulated in holoclone samples. However, one must be cognisant to the previously mentioned paucity of biological replicates in this study. Therefore, while this data provides a descriptive analysis of potential molecular profiles, additional studies are warranted to further validate these findings.

While shedding important light on how CSCs in PCa may be regulated by miRNAs, our results converge with the emerging theme that distinct miRNAs do regulate CSC properties. Our work to date has demonstrated that monoclonally-derived PC-3 and DU145 holoclones are capable of self-renewal, possess a high proliferative capacity, preferentially express embryonic stem-associated genes, and can generate xenograft tumours, which are phenotypically similar to those generated by differentiated parental cells. The unique genetic signature identified between parental and holoclone-derived cells provides data that further improves our understanding of mRNA-miRNA network interactions in PCa stem cell biology. Furthermore, we have identified holoclone-specific miRNAs that may represent potential therapeutic targeting of PCa stem cells, thereby potentially reversing some of the biological and clinical challenges associated with this rare subpopulation of cells in prostate cancer. Finally, using monoclonal cultivation of holoclones as an alternative approach to the generation of stem-like prostate cancer cells, our data identified holoclone-specific miRNAs that may represent potential therapeutic targets in prostate CSCs that may reverse, at least in part, some of the biological and clinical challenges associated with this rare subpopulation of cells.

## Materials and methods

### Prostate cell lines

PC-3, 22Rv1, DU145 and LNCaP were obtained from the ATCC (ATCC-LGC Standards, Teddington, Middlesex, UK). The PC-3 cell line was cultured in F12K Kaighn’s Modified Medium (Thermo-Fisher Scientific, Ireland) and 22Rv1 and LNCaP cell lines were cultured in RPMI (Sigma-Aldrich, St. Louis, MO, USA). The DU145 cell line was cultured in Eagle’s Minimum Essential Medium (Sigma) with 2 mM l-Glutamine (Sigma). All media were supplemented with 10% FBS (Sigma), penicillin streptomycin (5,000 U/mL penicillin, 5,000 U/mL streptomycin, Sigma). All cells were cultured in a 5% CO_2_ humidified atmosphere at 37 °C. Cell lines were screened for mycoplasma every 6 months using the MycoAlert mycoplasma detection kit (Lonza Group Ltd., Basel, Switzerland) according to manufacturers’ instructions.

### Flow cytometry

Cells (5 × 10^5^) were suspended in 100 μL PBA buffer (PBS, 0.1% NaN_3_, 0.1% BSA). Appropriate antibodies, APC mouse anti-human CD133 (Miltenyi Biotec, Germany); PE-Cy7 mouse anti-human CD44, PE mouse anti-human CD29, FITC-mouse anti-human CD49b (BD Biosciences, San Jose, CA, USA) were added to each cell sample and incubated in the dark for 20 min. A further 100 μL PBA was added; cells were pelleted to remove unbound antibody and re-suspended in 200 μL PBA buffer. The samples were then acquired on a Dako CyAn ADP flow cytometer (Beckman Coulter, Brea, CA, USA).

### High-salt agar

A sterile 1% molecular grade agarose (Sigma) and NaCl (Sigma) solution was prepared, added to petri dishes (Corning Incorporated, Corning, NY, USA) and allowed to solidify. Media (15 mL) was added to the agar surface with 1 × 10^6^ cells and incubated under standard conditions. Media was replaced at 7-day intervals and observed for colony formation through microscopic interrogation. Media (7–10 mL) was carefully removed from the periphery of the plates every 7 days and replaced with fresh, pre-warmed media until the end of the experiment. The media used in these experiments was the same as that used for standard culture of the cells.

### Colony forming assay

DU145 and PC-3 PCa cell lines were cultured and harvested at 70–80% confluence according to standard laboratory protocols. Cell pellets were collected from DU145 and PC-3 cell lines to serve as controls from which total RNA was isolated. The remaining cells (5 × 10^5^) were re-suspended in sterile PBS containing 1% BSA. Using a MoFlo XDP high speed cell sorter (Beckman Coulter, USA), a single cell from both cell lines was seeded separately into each well, in triplicate, of 10 round-bottomed 96-well plates (Nunc, Germany) containing culture media. Two days following plating, the 96-well plates were examined using light microscopy and wells containing only one viable cell were identified. Seven days following plating, colonies derived from single cells were classified as holo-, mero-, and paraclones based on cell morphology^12^. Images were acquired and holoclones were harvested at 14 days. At this time-point, wells containing holoclones were washed trypsinised and holoclones from all wells from each replicate were pooled together. Resulting pellets were stored for subsequent analysis. The media used in these experiments was the same as that used for standard culture of the cells.

### Gene expression analysis

Total RNA was isolated using TRI Reagent (Molecular Research Centre, USA) according to manufacturer’s instructions. cDNA was synthesised from 2 µg RNA using the High Capacity cDNA Reverse Transcription Kit (Applied Biosystems, Foster City, CA, USA) according to manufacturers’ instructions. The thermal cycling conditions were as follows; 25 °C for 10 min, 37 °C for 120 min and 85 °C for 5 min. Real-time qRT-PCR was performed using the 7,500 Fast Real-Time PCR platform (Applied Biosystems, Foster City, CA, USA) and relative quantification (RQ) values were determined based on 2^−∆∆Ct^ using DataAssist software (Applied Biosystems). Primer information is provided in Table 1.

**Table 1:** qRT-PCR primer information.

Gene symbol	Gene name	TaqMan assay ID
ALDH1A1	Aldehyde dehydrogenase	Hs000946916_m1
SOX2	SRY-box2	Hs01053049_s1
POUF51	POU class 5 homeobox1	Hs00999634_gH
NANOG	Nanog Homeobox	Hs04260366_g1
GAPDH	Glyceraldehydes-3-phosphate dehydrogenase	Hs02758991_g1
CD44	CD44 molecule	Hs01075861_m1
PROM1	Prominin 1/CD133	Hs01009250_m1
ITGB1	Integrin beta 1	Hs01009250_m1
ITGA2	Integrin alpha 2	Hs00158127_m1
MET	Met proto-oncogene	Hs01565584_m1
CD24	CD24 molecule	Hs00273561_s1
hsa-miR-15a	microRNA 15a	000389
hsa-miR-21	microRNA 21	000397
hsa-miR16-1	microRNA 16-1′	002420
hsa-miR-125b	microRNA 125b	000449
hsa-miR-20a	microRNA 20a	000580
hsa-miR-34a	microRNA 34a	000426
hsa-miR-222	microRNA 222	002276
hsa-miR-221	microRNA 221	000524
hsa-miR126	microRNA 126	002228
RNU24	Small nucleolar RNA C/D box 24	001001
hsa-miR-331	microRNA 331	000545
hsa-miR-200b	microRNA 200b	002251
hsa-miR-101	microRNA 101	002253
hsa-miR-146a	microRNA 146a	000468
hsa-miR-141	microRNA 141	000463
hsa-miR-330	microRNA 330	000544

### microRNA (miRNA) analysis

cDNA was synthesised from 10 ng total RNA using the TaqMan microRNA Reverse Transcription Kit (Applied Biosystems) according to manufacturers’ instructions. A master mix containing target primers (Table 1) and endogenous control (RNU24) was prepared for each individual miRNA. Thermal cycling conditions used were as follows; 16 °C for 30 min, 42 °C for 30 min, and 85 °C for 5 min. Real-time qRT-PCR was performed and analysed as per gene expression analysis.

### *In vivo study*

All animal experiments were performed in compliance with the Irish Department of Health and Children regulations (Licence: B100/3250) and approved by the Trinity College Dublin BioResource Ethical Review Board (Ref: 121108). PC-3 and DU145 (3 × 10^3^) parental and holoclone cells (n = 6 per group), were injected subcutaneously above the right hind-limb of 9–10 week old male NOD/SCID mice (strain NOD.CB17-Prkdcscid/NCrHsd mice; Harlan, Bicester, UK) using a Ham’s F12 (Sigma) and matrigel (BD Biosciences) mix. The injection site was shaved prior to injection and ear punches were applied in order to identify the mice. Measurement of tumour diameters or volumes is a commonly used tool in experimental tumour models to quantitate the effects of experimental manipulations on tumour growth by comparing control and treated groups of mice and rats. Typically, average tumour diameters at one or more discrete time points are compared using a statistical test, while survival curves of tumour-bearing animals are generated as a function of time and compared by statistical methods. Tumour volume was calculated every 4–5 days using callipers and the modified ellipsoid formula ½ (Length × Width^2^). In addition to quantifying differences in tumour volumes between parent cells and holoclones-derived from same, areas under the curve (AUC) were also used as an alternative method of assessing tumour growth curves^80^, permitting quantitation of tumour growth not generally measured using traditional methods. Using GraphPad Prism software (La Jolla, CA), area under curve (AUC) was calculated for DU145 and PC-3 parent cells and holoclones based on tumour volumes at each time-point. When derivative tumours had reached a pre-defined ethical limit, mice were euthanized by cervical dislocation. A portion of the harvested tumour was snap-frozen in liquid nitrogen, while the remainder of the tumour was used for histological analysis. Tissue was fixed using 10% neutral buffered formalin (max. 8 h), paraffin embedded and sectioned according to standard laboratory protocols. Prior to paraffin embedding, tissue was cleared through soaking in 50:50 toluene and EtOH. Sections were stained using H&E and reviewed by a pathologist.

### Immunohistochemistry (IHC)

Tissue sections (5 µm) were cut onto charged slides and baked overnight at 65 °C. A protocol was designed on the Discovery XT machine (Roche Ventana, Arizona, USA) according to optimum antibody conditions (Table 2). Unique barcoded labels corresponding to the specific protocol were created and attached to the charged slides. The slides were inserted into the auto-stainer along with the required reagents. Upon protocol completion, the slides were removed and washed with Dako 1× wash buffer and distilled water to remove residual liquid coverslip. The slides were then placed in haematoxylin for 5–6 s and subsequently rinsed in running water to remove excess dye. The slides were placed in 70% EtOH for 1 min, 100% EtOH for 1–2 min, xylene for 2 min and cover-slipped for analysis.

**Table 2:** IHC antibodies.

	Incubation time (min)	Optimal dilution	Company	Ref. code
**1° antibody**				
Anti-ERG Rabbit mAB	32	Neat	Roche	EPR3864
Anti-Ki-67 Mouse mAB	32	1:80	Dako	IS62630-2
Anti-CCND1 Rabbit mAB	24	Neat	Dako	IS08330-2
Anti-Cytokeratin (Cam5.2) Rabbit mAB	16	Neat	Roche	790-4555
Anti-E-cadherin Mouse mAB	32	1:50	Invitrogen	18-0223
Anti-CD34 Mouse mAB	32	1:50	Dako	IR63261-2
Anti-Vimentin Mouse mAB	16	Neat	Dako	IR63061-2
**2° antibody**				
OmniMap anti-Rabbit HRP	20	Neat	Roche	760-4311
Universal Secondary Antibody MultiLink	20	Neat	Dako	E045301-2

### Next-generation sequencing (NGS)

NGS of small (single read) and long ncRNA (paired-end) repertoires of clonally-derived holoclones and their derivative tumour xenografts was performed by Clinical Genomics (Mount Sinai Hospital, Toronto, Canada) using the Illumina HiSeq 2500 platform (Illumina, San Diego, CA, USA). The Illumina Cluster Station isothermally amplified DNA on a flow cell surface to generate clusters, each of which contained 500–1,000 clonal copies of a single template molecule. In the case of paired-end reads, following completion of the first read, the clusters were modified to regenerate the template for the paired read. The same clusters were then sequenced using a second primer to generate the second read. The benefit of utilising paired-end reads for sequencing of mRNA and long ncRNAs is that the paired end nature allows more accurate alignments.

### Analysis of sequencing data

Global gene expression was analysed among cellular parent and holoclone samples (PC-3 and DU145) and their respective derivative murine tumours on the Illumina HiSeq 2500 system. In total 8 samples were sequenced on two paired-end HiSeq 2500 lanes. The data passed all primary analyses and any sequencing errors were identified and filtered from the final dataset. Reads were quality-trimmed using Cutadapt and Sickle^81–83^. Filtered reads were mapped to the Ensembl Human reference genome (build GRCh38, release 76) and sequences were annotated based on their overlap with publicly available mRNA transcripts. Short RNAs were mapped using Bowtie, while long RNAs were mapped using the splice-aware aligner Tophat, allowing reads that span splice junctions to be properly mapped^84,85^. Read counts across the transcriptome were subsequently used to determine differential expression patterns between samples. Read counts were calculated across all transcripts using HTSeq^86^. Transcripts which received low average counts (≤ 100) across samples were excluded to promote evidence-based results. Differential expression analysis and log2 fold-changes were calculated internally by edgeR^87^. The number of reads that mapped to loci of known transcripts were used to calculate abundances, and therefore infer the expression levels of those transcripts within a given sample. These loci and other transcript information were provided by Ensembl for the longer RNAs, while miRBase provided the annotation for the small RNA analysis. The lack of biological replicates meant the results were restricted to a descriptive analysis of the two cell lines and various conditions under study. As a result, individual differential expression test results were limited in their utility, and additional filtering of results was necessary. To identify the most biologically relevant differences between parent and holoclones, the results from PC-3 and DU145 analysis were intersected, with the overlapping genes postulated to represent the most pertinent molecular alterations.

For Network of miRNA: Target interactions (MTIs), genes and miRNAs identified as being dysregulated were further investigated to identify interactions using databases of miRNA: Target Interactions (MTIs) and queried to form lists of interacting miRNA-gene pairs, which in turn were used to create integrated networks. In total, five databases were used. Three of these, miRWalk, miRTarBase and miRecords, include manually curated datasets of experimentally validated interactions. The remainder (TargetScan and miRTar) comprise lists of computationally-predicted interactions. The results from each separate database were concatenated and visualised as directed acyclic graphs.

## Supplementary information

Supplementary Information 1

## References

[1] Bray F (2018). Global cancer statistics 2018: GLOBOCAN estimates of incidence and mortality worldwide for 36 cancers in 185 countries. CA Cancer J. Clin..

[2] Salinas CA, Tsodikov A, Ishak-Howard M, Cooney KA (2014). Prostate cancer in young men: An important clinical entity. Nat. Rev. Urol..

[3] Inamura K (2018). Prostatic cancers: Understanding their molecular pathology and the 2016 WHO classification. Oncotarget.

[4] Gandhi J (2018). The molecular biology of prostate cancer: Current understanding and clinical implications. Prostate Cancer Prostatic Dis..

[5] Hughes C, Murphy A, Martin C, Sheils O, O'Leary J (2005). Molecular pathology of prostate cancer. J. Clin. Pathol..

[6] Batlle E, Clevers H (2017). Cancer stem cells revisited. Nat. Med..

[7] Maitland NJ, Collins AT (2010). Cancer stem cells—A therapeutic target?. Curr. Opin. Mol. Ther..

[8] Packer JR, Maitland NJ (1863). The molecular and cellular origin of human prostate cancer. Biochim. Biophys. Acta.

[9] Dexter TM, Allen TD, Lajtha LG (1977). Conditions controlling the proliferation of haemopoietic stem cells in vitro. J. Cell Physiol..

[10] Foster CS, Dodson A, Karavana V, Smith PH, Ke Y (2002). Prostatic stem cells. J. Pathol..

[11] Hall PA, Watt FM (1989). Stem cells: The generation and maintenance of cellular diversity. Development.

[12] Barrandon Y, Green H (1987). Three clonal types of keratinocyte with different capacities for multiplication. Proc. Natl. Acad. Sci. USA.

[13] Jordan CT, Guzman ML, Noble M (2006). Cancer stem cells. N. Engl. J. Med..

[14] Mackenzie IC (2005). Retention of stem cell patterns in malignant cell lines. Cell Prolif..

[15] Reya T, Morrison SJ, Clarke MF, Weissman IL (2001). Stem cells, cancer, and cancer stem cells. Nature.

[16] Skvortsov S, Skvortsova II, Tang DG, Dubrovska A (2018). Concise review: Prostate cancer stem cells: Current understanding. Stem Cells.

[17] Rybak AP, Bristow RG, Kapoor A (2015). Prostate cancer stem cells: Deciphering the origins and pathways involved in prostate tumorigenesis and aggression. Oncotarget.

[18] Mei W (2019). The contributions of prostate cancer stem cells in prostate cancer initiation and metastasis. Cancers (Basel).

[19] Li JJ, Shen MM (2019). Prostate stem cells and cancer stem cells. Cold Spring Harb. Perspect. Med..

[20] Al-Hajj M, Wicha MS, Benito-Hernandez A, Morrison SJ, Clarke MF (2003). Prospective identification of tumorigenic breast cancer cells. Proc. Natl. Acad. Sci. USA.

[21] Singh SK (2003). Identification of a cancer stem cell in human brain tumors. Cancer Res..

[22] O'Brien CA, Pollett A, Gallinger S, Dick JE (2007). A human colon cancer cell capable of initiating tumour growth in immunodeficient mice. Nature.

[23] Li C, Lee CJ, Simeone DM (2009). Identification of human pancreatic cancer stem cells. Methods Mol. .Biol.

[24] Bertolini G (2009). Highly tumorigenic lung cancer CD133+ cells display stem-like features and are spared by cisplatin treatment. Proc. Natl. Acad. Sci. USA.

[25] Cojoc M (2015). Aldehyde dehydrogenase is regulated by beta-catenin/TCF and promotes radioresistance in prostate cancer progenitor cells. Cancer Res..

[26] Kyriakopoulos CE (2018). Chemohormonal therapy in metastatic hormone-sensitive prostate cancer: long-term survival analysis of the randomized phase III E3805 CHAARTED trial. J. Clin. .Oncol.

[27] Olszewski WL, Moscicka M, Zolich D, Machowski Z (2005). Human keratinocyte stem cells survive for months in sodium chloride and can be successfully transplanted. Transpl. Proc..

[28] Zhang K, Waxman DJ (2010). PC3 prostate tumor-initiating cells with molecular profile FAM65Bhigh/MFI2low/LEF1low increase tumor angiogenesis. Mol. Cancer.

[29] Wintzell M (2012). Repeated cisplatin treatment can lead to a multiresistant tumor cell population with stem cell features and sensitivity to 3-bromopyruvate. Cancer Biol. Ther..

[30] Li H, Chen X, Calhoun-Davis T, Claypool K, Tang DG (2008). PC3 human prostate carcinoma cell holoclones contain self-renewing tumor-initiating cells. Cancer Res..

[31] Locke M, Heywood M, Fawell S, Mackenzie IC (2005). Retention of intrinsic stem cell hierarchies in carcinoma-derived cell lines. Cancer Res..

[32] Pfeiffer MJ, Schalken JA (2010). Stem cell characteristics in prostate cancer cell lines. Eur. Urol..

[33] Patrawala L (2006). Highly purified CD44+ prostate cancer cells from xenograft human tumors are enriched in tumorigenic and metastatic progenitor cells. Oncogene.

[34] Tan L, Sui X, Deng H, Ding M (2011). Holoclone forming cells from pancreatic cancer cells enrich tumor initiating cells and represent a novel model for study of cancer stem cells. PLoS ONE.

[35] Jeter CR (2009). Functional evidence that the self-renewal gene NANOG regulates human tumor development. Stem Cells.

[36] Liu TJ (2013). CD133+ cells with cancer stem cell characteristics associates with vasculogenic mimicry in triple-negative breast cancer. Oncogene.

[37] Harper LJ, Piper K, Common J, Fortune F, Mackenzie IC (2007). Stem cell patterns in cell lines derived from head and neck squamous cell carcinoma. J. Oral Pathol. Med..

[38] Ferrand A, Sandrin MS, Shulkes A, Baldwin GS (2009). Expression of gastrin precursors by CD133-positive colorectal cancer cells is crucial for tumour growth. Biochim. Biophys. Acta.

[39] Tang DG (2007). Prostate cancer stem/progenitor cells: Identification, characterization, and implications. Mol. Carcinog..

[40] Russell PJ (2015). Establishing prostate cancer patient derived xenografts: Lessons learned from older studies. Prostate.

[41] Liu X (2015). Systematic dissection of phenotypic, functional, and tumorigenic heterogeneity of human prostate cancer cells. Oncotarget.

[42] Rajasekhar VK, Studer L, Gerald W, Socci ND, Scher HI (2011). Tumour-initiating stem-like cells in human prostate cancer exhibit increased NF-kappaB signalling. Nat. Commun..

[43] Tokar EJ, Ancrile BB, Cunha GR, Webber MM (2005). Stem/progenitor and intermediate cell types and the origin of human prostate cancer. Differentiation.

[44] Stone KR, Mickey DD, Wunderli H, Mickey GH, Paulson DF (1978). Isolation of a human prostate carcinoma cell line (DU 145). Int. J. Cancer.

[45] Sramkoski RM (1999). A new human prostate carcinoma cell line, 22Rv1. Vitro Cell Dev. Biol. Anim..

[46] Kaighn ME, Narayan KS, Ohnuki Y, Lechner JF, Jones LW (1979). Establishment and characterization of a human prostatic carcinoma cell line (PC-3). Invest. Urol..

[47] Horoszewicz JS (1983). LNCaP model of human prostatic carcinoma. Cancer Res..

[48] Pulukuri SM (2005). RNA interference-directed knockdown of urokinase plasminogen activator and urokinase plasminogen activator receptor inhibits prostate cancer cell invasion, survival, and tumorigenicity in vivo. J. Biol. Chem..

[49] Gallagher MF (2015). Enhanced regulation of cell cycle and suppression of osteoblast differentiation molecular signatures by prostate cancer stem-like holoclones. J. Clin. Pathol..

[50] Lynam-Lennon N (2017). MicroRNA-17 is downregulated in esophageal adenocarcinoma cancer stem-like cells and promotes a radioresistant phenotype. Oncotarget.

[51] Patrawala L, Calhoun-Davis T, Schneider-Broussard R, Tang DG (2007). Hierarchical organization of prostate cancer cells in xenograft tumors: the CD44+alpha2beta1+ cell population is enriched in tumor-initiating cells. Cancer Res..

[52] Matsuda S (2014). Cancer stem cells maintain a hierarchy of differentiation by creating their niche. Int. J. Cancer.

[53] Wei C, Guomin W, Yujun L, Ruizhe Q (2007). Cancer stem-like cells in human prostate carcinoma cells DU145: The seeds of the cell line?. Cancer Biol. Ther..

[54] Rochat A, Kobayashi K, Barrandon Y (1994). Location of stem cells of human hair follicles by clonal analysis. Cell.

[55] Pellegrini G (1999). Location and clonal analysis of stem cells and their differentiated progeny in the human ocular surface. J. Cell Biol..

[56] Papini S (2003). Isolation and clonal analysis of human epidermal keratinocyte stem cells in long-term culture. Stem Cells.

[57] Zhou ZH (2009). A novel approach to the identification and enrichment of cancer stem cells from a cultured human glioma cell line. Cancer Lett..

[58] Collins AT, Berry PA, Hyde C, Stower MJ, Maitland NJ (2005). Prospective identification of tumorigenic prostate cancer stem cells. Cancer Res..

[59] Beaver CM, Ahmed A, Masters JR (2014). Clonogenicity: holoclones and meroclones contain stem cells. PLoS ONE.

[60] Moltzahn F, Thalmann GN (2013). Cancer stem cells in prostate cancer. Transl. Androl. Urol..

[61] Hess DA (2004). Functional characterization of highly purified human hematopoietic repopulating cells isolated according to aldehyde dehydrogenase activity. Blood.

[62] Ginestier C (2007). ALDH1 is a marker of normal and malignant human mammary stem cells and a predictor of poor clinical outcome. Cell Stem Cell.

[63] Corti S (2006). Identification of a primitive brain-derived neural stem cell population based on aldehyde dehydrogenase activity. Stem Cells.

[64] Doherty RE, Haywood-Small SL, Sisley K, Cross NA (2011). Aldehyde dehydrogenase activity selects for the holoclone phenotype in prostate cancer cells. Biochem. Biophys. Res. Commun..

[65] Bissell MJ, Labarge MA (2005). Context, tissue plasticity, and cancer: are tumor stem cells also regulated by the microenvironment?. Cancer Cell.

[66] Zhou Y (2017). Isolation and identification of cancer stem cells from PC3 human prostate carcinoma cell line. Int. J. Clin. Exp. Pathol..

[67] Liu C (2017). MicroRNA-141 suppresses prostate cancer stem cells and metastasis by targeting a cohort of pro-metastasis genes. Nat. Commun..

[68] Hsieh IS (2013). MicroRNA-320 suppresses the stem cell-like characteristics of prostate cancer cells by downregulating the Wnt/beta-catenin signaling pathway. Carcinogenesis.

[69] Jin M (2014). miRNA-128 suppresses prostate cancer by inhibiting BMI-1 to inhibit tumor-initiating cells. Cancer Res..

[70] Huang S (2012). miR-143 and miR-145 inhibit stem cell characteristics of PC-3 prostate cancer cells. Oncol. Rep..

[71] Sadeghi M (2016). MicroRNA and transcription factor gene regulatory network analysis reveals key regulatory elements associated with prostate cancer progression. PLoS ONE.

[72] Tao ZQ (2016). Role of microRNA in prostate cancer stem/progenitor cells regulation. Eur. Rev. Med. Pharmacol. Sci..

[73] Rane JK (2015). MicroRNA expression profile of primary prostate cancer stem cells as a source of biomarkers and therapeutic targets. Eur. Urol..

[74] Wang D, Lu G, Shao Y, Xu D (2018). MiR-182 promotes prostate cancer progression through activating Wnt/beta-catenin signal pathway. Biomed. Pharmacother..

[75] Baumann B (2019). Association of high miR-182 levels with low-risk prostate cancer. Am. J. Pathol..

[76] Guan H (2017). MicroRNA-744 promotes prostate cancer progression through aberrantly activating Wnt/beta-catenin signaling. Oncotarget.

[77] Zhang M, Li H, Zhang Y, Li H (2019). Oncogenic miR-744 promotes prostate cancer growth through direct targeting of LKB1. Oncol. Lett..

[78] Vlachos IS (2015). DIANA-miRPath v3.0: Deciphering microRNA function with experimental support. Nucleic Acids Res..

[79] Liu C (2012). Distinct microRNA expression profiles in prostate cancer stem/progenitor cells and tumor-suppressive functions of let-7. Cancer Res..

[80] Duan F (2012). Area under the curve as a tool to measure kinetics of tumor growth in experimental animals. J. Immunol. Methods.

[81] Joshi, N. A. & Fass, J. N. Sickle: A sliding-window, adaptive, quality-based trimming tool for FastQ files (Version 1.33) [Software]. Available at https://github.com/najoshi/sickle (2011).

[82] Martin M (2011). Cutadapt Removes Adapter Sequences from High-Throughput Sequencing Reads. EMBnet J..

[83] Chen C, Khaleel SS, Huang H, Wu CH (2014). Software for pre-processing Illumina next-generation sequencing short read sequences. Source Code Biol. Med..

[84] Langmead B, Trapnell C, Pop M, Salzberg SL (2009). Ultrafast and memory-efficient alignment of short DNA sequences to the human genome. Genome Biol..

[85] Trapnell C, Pachter L, Salzberg SL (2009). TopHat: Discovering splice junctions with RNA-Seq. Bioinformatics.

[86] Anders S, Pyl PT, Huber W (2015). HTSeq—A Python framework to work with high-throughput sequencing data. Bioinformatics.

[87] Robinson MD, McCarthy DJ, Smyth GK (2010). edgeR: A Bioconductor package for differential expression analysis of digital gene expression data. Bioinformatics.

