# Supplementary material for: Prostate cancer-derived holoclones: a novel and effective model for evaluating cancer stemness
Source: Sci Rep. 2020 Jul 9;10:11329. doi: 10.1038/s41598-020-68187-9 (PMC7347552; doi:10.1038/s41598-020-68187-9)
Supplement: Supplementary file 1 — Supplementary Information 1 [file 41598_2020_68187_MOESM1_ESM.pdf]

Supplementary information

Prostate cancer-derived holoclones: a novel and effective model for evaluating cancer stemness

Louise Flynn, Martin P. Barr, Anne-Marie Baird, Paul Smyth, Orla M. Casey, Gordon Blackshields, John Greene, Stephen R. Pennington, Emily Hams, Padraic G. Fallon, John O'Leary, Orla Sheils and Stephen P. Finn

Stem cell markers are expressed in prostate cancer cell lines

A panel of four cell lines (DU145, PC-3, 22Rv1 and LNCaP) were assessed for a specific stem cell marker signature (integrin α2β1hi, CD44+ and CD133+) using FACS (n=1; Supplementary Fig. 1). This panel was identified through a concise review of relevant literature published in peer reviewed journals [27]. This signature was differentially expressed across the cell line panel (Supplementary Table S1 and Supplementary Fig. 1). While integrin α2β1 and CD44 were highly expressed in all 4 cell lines, a larger percentage of LNCaP cells were positive for CD133 (16.9%).

(i) DU145

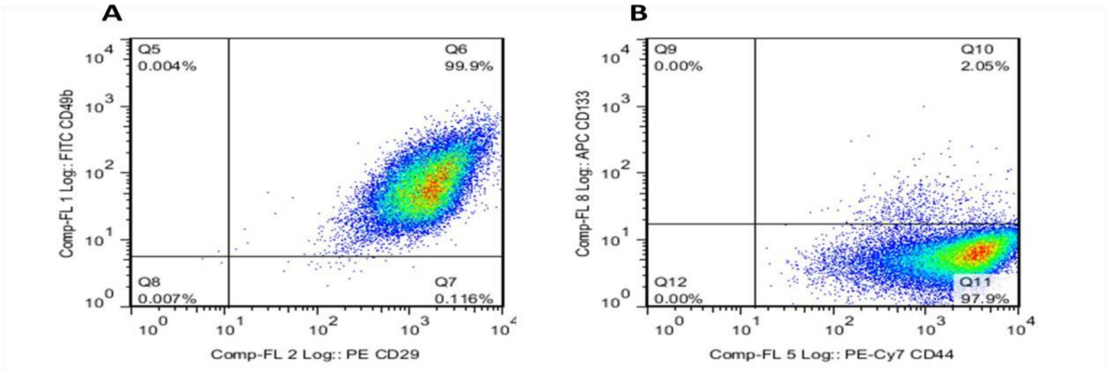

(ii) PC-3

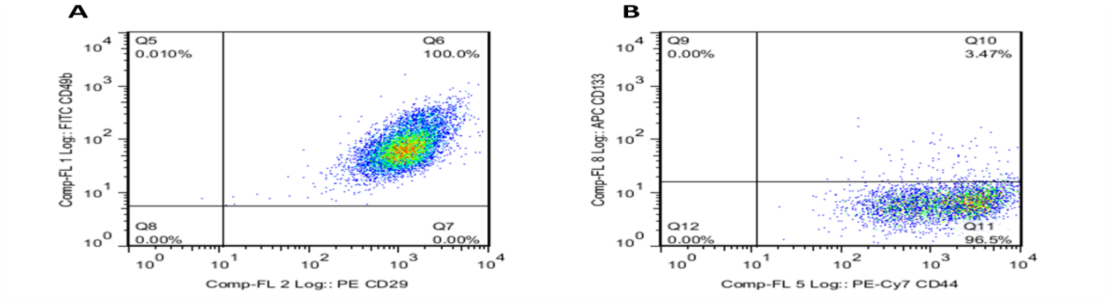

(iii) 22Rv1

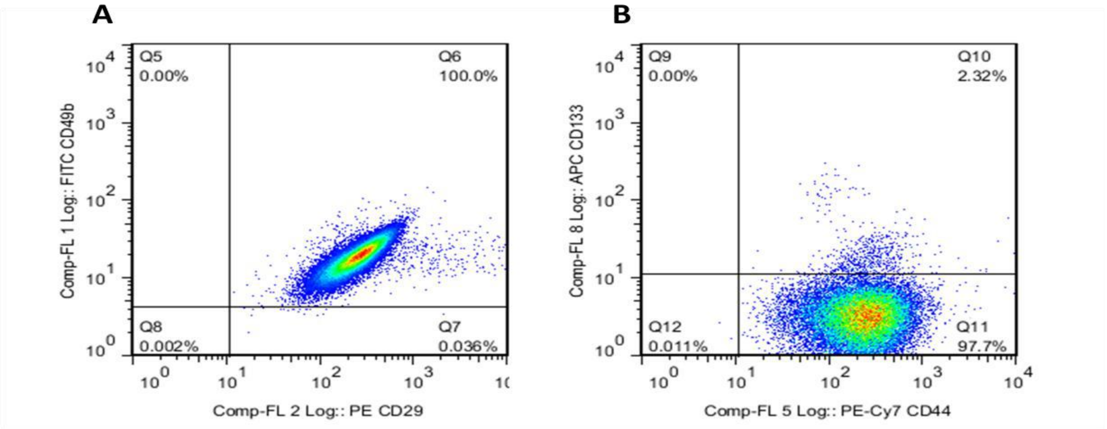

(iv) LNCaP

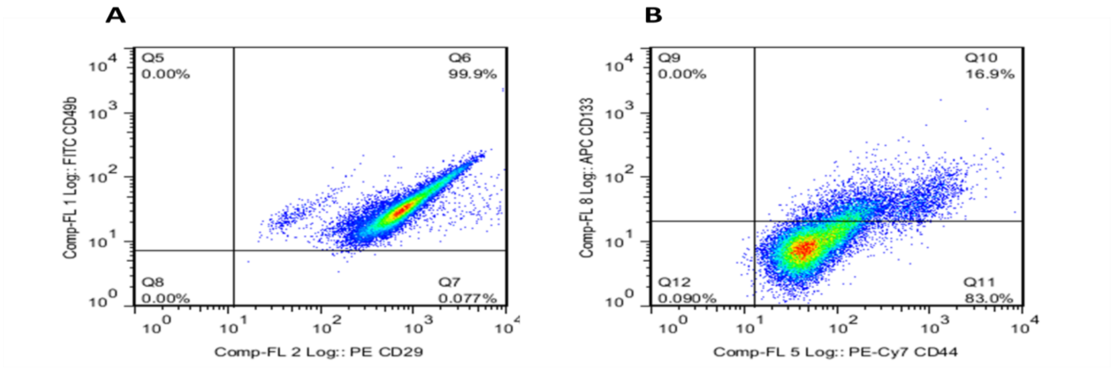

Supplementary Figure 1: Flow cytometry dot plots for (i) DU145, (ii) PC-3, (iii) 22Rv1 and (iv) LNCaP, demonstrating (A) integrinα2β1 and (B) CD44/CD133 expression. (n=1)

PC-3 and DU145 Cells Exhibit Differential Propensities to Efficiently Generate Heterogeneous Colonies

PC-3 and DU145 cells were found to exhibit disparate abilities to generate phenotypically plastic colonies. Across three biological replicates (n=15 plates/replicate), 20% of the colonies produced by PC-3 cells comprised of holoclones (Fig. S2A), while the majority of colonies produced were meroclones (64%). Paraclones generated at a rate similar to holoclones (16%). Conversely, under the same parameters, 43% of the total colonies produced by DU145 cells constituted holoclones (Fig. S2A). Furthermore, meroclones were produced at a rate similar to holoclones (44%) while paraclones comprised 13% of the colony total. PC-3 cells demonstrated the ability to generate more Colony Forming Units compared to DU145 cells for all types of clones formed (Fig. S2B).

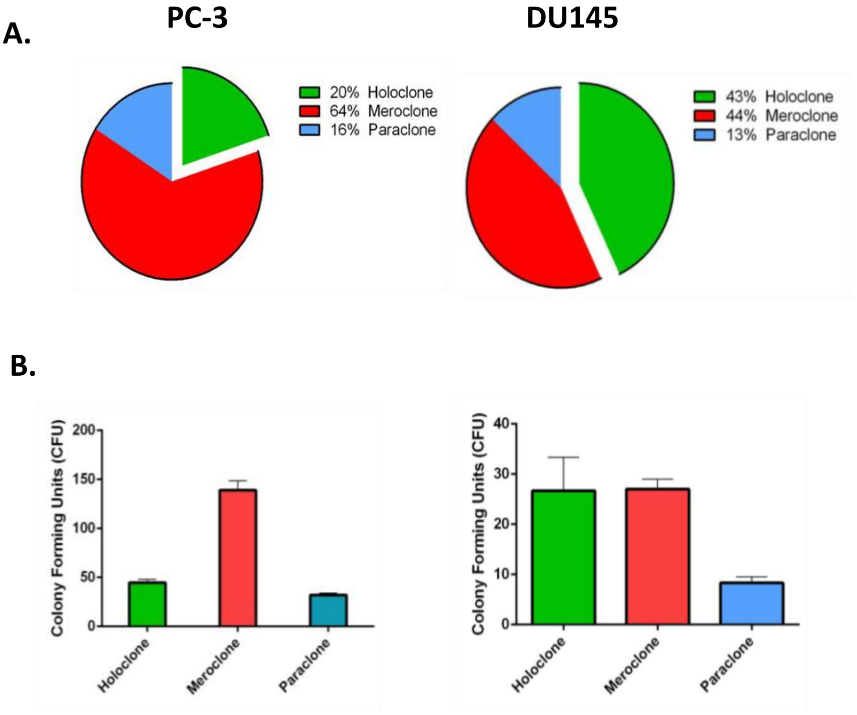

Supplementary Figure 2: Colony forming efficiency.

(A) Percentage of distinct colonies produced by PC-3 and DU145 cells expressed as a proportion of total colonies produced across multiple experiments (n=3, 15 plates/experiment). (B) Colony forming units for PC-3 and DU145 cells across multiple experiments. Data represented as Mean ± SEM (n=3, 15 plates/experiment).

Holoclones only can regenerate all colony morphologies

Dissociation of distinct PC-3 and DU145 colonies, expansion within a 12-well dish and subsequent resubmission to monoclonal cultivation demonstrated that only holoclones had the capability to regenerate all colony morphologies (Fig. S3). Meroclones were found to regenerate only meroclones and paraclones, while paraclones exhibited modest ability to regenerate their own morphology (Fig. S3). Furthermore, dissociation and re-plating of PC-3 and DU145 holoclones resulted in a higher frequency of holoclone generation than observed in first-round plating.

(i) PC-3

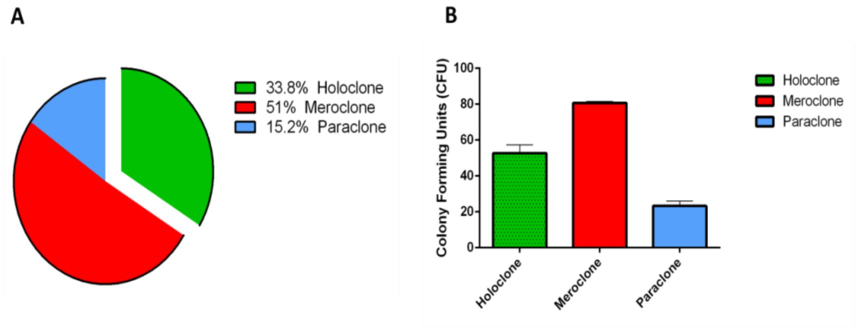

(ii) DU145

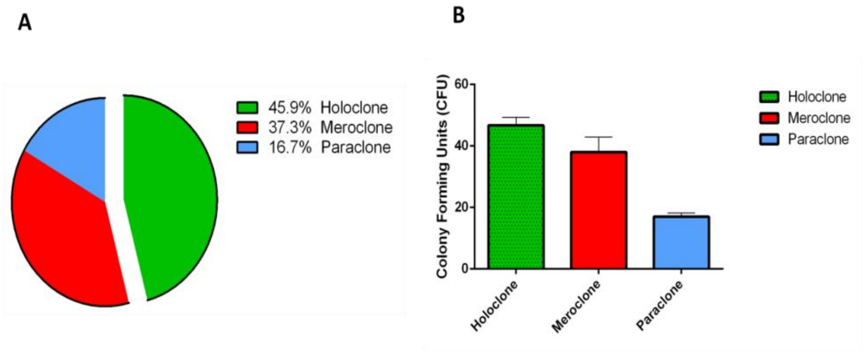

Supplementary Figure 3: Only holoclones had the ability to regenerate all colony morphologies.

PC-3 (i) and DU145 (ii) re-plated holoclones generated a larger number of holoclones than parental cells. (A) Percentage of distinct colonies produced by cells expressed as a proportion of total colonies produced across multiple experiments (n=3, 15 plates/experiment). (B) Colony forming units of cells across multiple experiments
(n=3, 5 plates per experiment). Data represented as Mean ± SEM.

Prostatospheres display altered expression of a stem-associated gene panel

The ability of PC-3 and DU145 cells to generate three-dimensional prostatospheres was assayed by culture in serum-free medium supplemented with EGF, bFGF, BSA and insulin[37]. Briefly, Dulbecco's Modified Eagles Medium/Nutrient Mixture F-12 Ham (Sigma Aldrich, St. Louis, MO, USA) was supplemented with 0.4% BSA (Sigma Aldrich, St. Louis, MO, USA), 1% penicillin streptomycin (Sigma Aldrich, St. Louis, MO, USA), 2.5 mg insulin (Gibco®, Life Technologies, Biosciences, Ireland), 5 µg recombinant human fibroblast growth factor (FGF) (Cell Signalling, MA, USA), and 10 µg recombinant human epidermal growth factor (Peprotech, New Jersey, USA). Cells were seeded at low density (1×10^4) in a T25 flask and observed every 2-3 days for sphere formation. Neither PC-3 nor DU145 cells generated morphologically unique spheres. Both cell lines displayed growth patterns equivalent to culture in standard medium. However, cells were harvested at 14 days and their stem phenotype assessed by examining the expression of the stem-associated gene panel. Samples were normalised to the relevant parental cell line. OCT4/POU5F1 ($p= 0.01$), NANOG ($p= 0.002$), and SOX2 ($p= 0.001$) were found upregulated in DU145 cells grown in stem cell medium (Fig S4A). CD44 and CD133 were also upregulated but did not reach significance. ALDH1 was found to be downregulated in DU145 'sphere' cells. All stem-associated genes were upregulated in PC-3 cells cultured in stem cell medium but did not reach significance (Fig. S4B).

(A) DU145

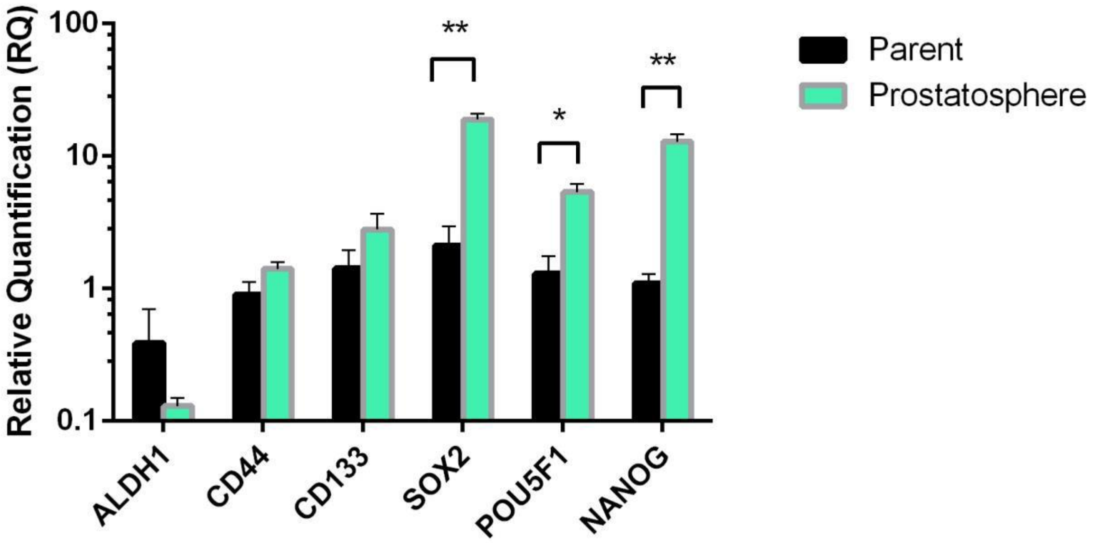

(B) PC-3

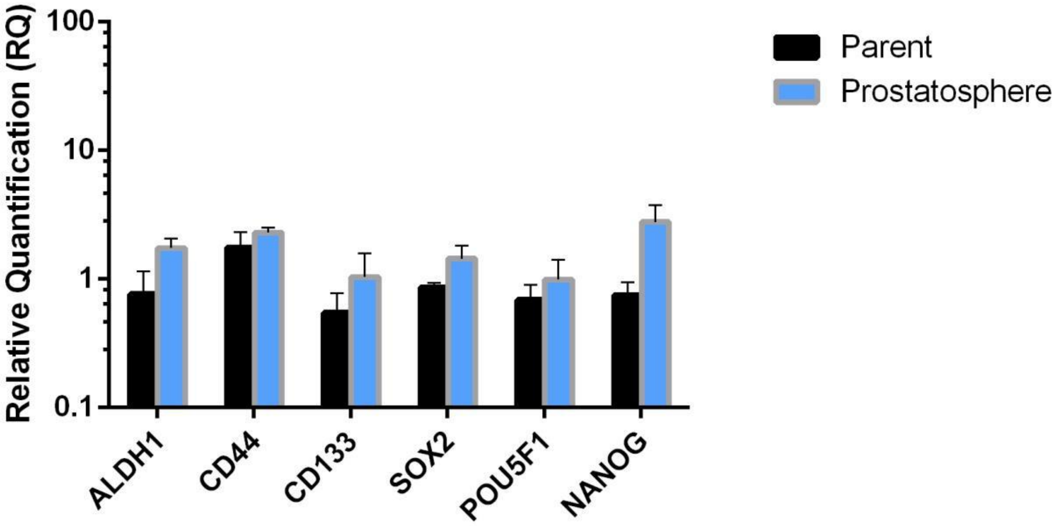

Supplementary Figure 4: Stemness gene expression analysis in (A) DU145 and (B) PC-3 ‘prostatospheres’ (cells cultured in stem cell medium) (n=3).
Relative quantification (RQ) of change in expression of stem-associated genes in holoclones normalised to parental controls. Data represented as Mean ± SEM (*p<0.05, **p<0.01, unpaired Student’s two-tailed *t* test, n=3).

Validation of selected miRNAs in PC-3 and DU145 control (parental) and holoclone derived cells

A.

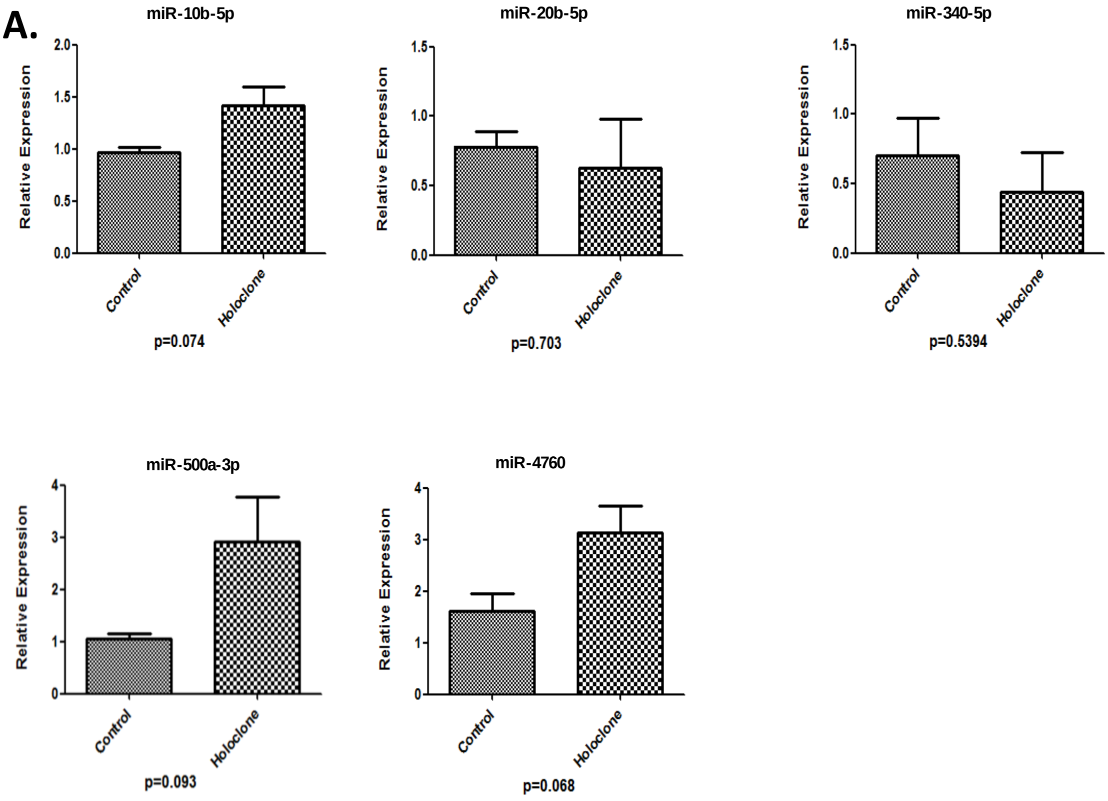

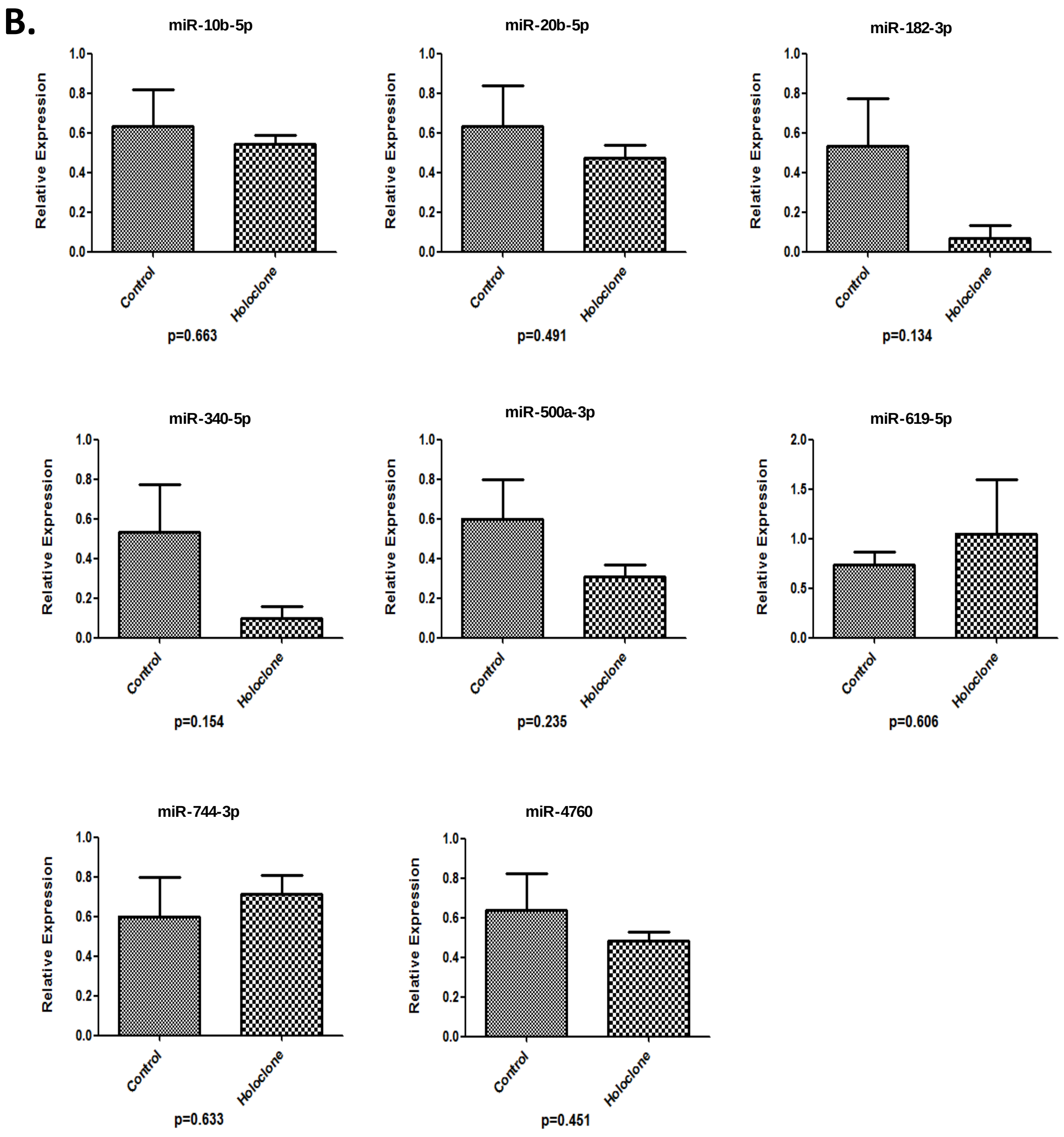

Supplementary Figure 5: Validation of selected miRNAs in PC-3 (A) and DU145 (B) control (parental) and holoclone derived cells.

(Relative quantification of change in expression of stem-associated genes in holoclones normalised to parental control). Data represented as Mean ± SEM. (unpaired student's two-tailed *t* test, n=3).

Network of miRNA: target interactions (MTIs)

A.

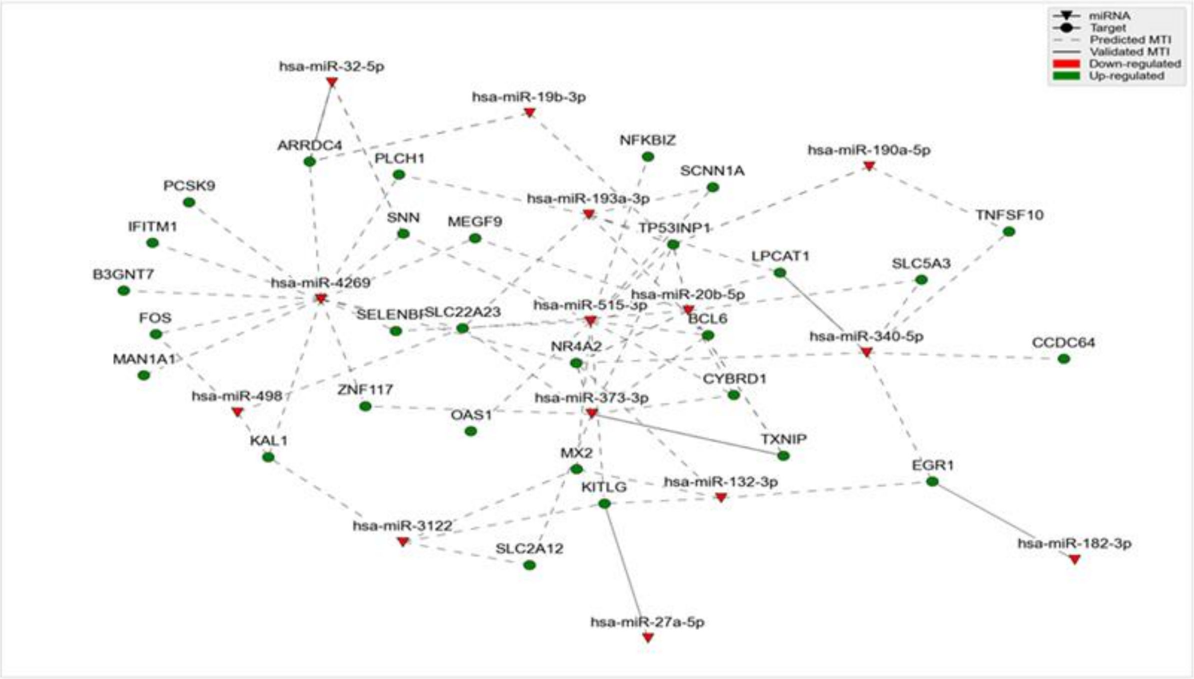

B.

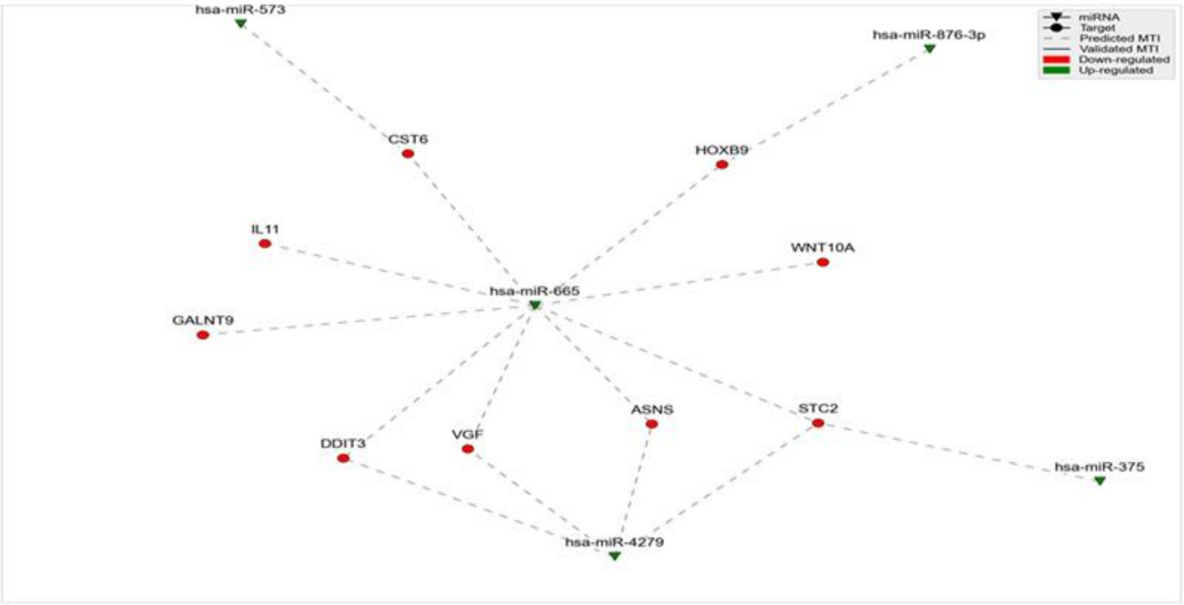

C.

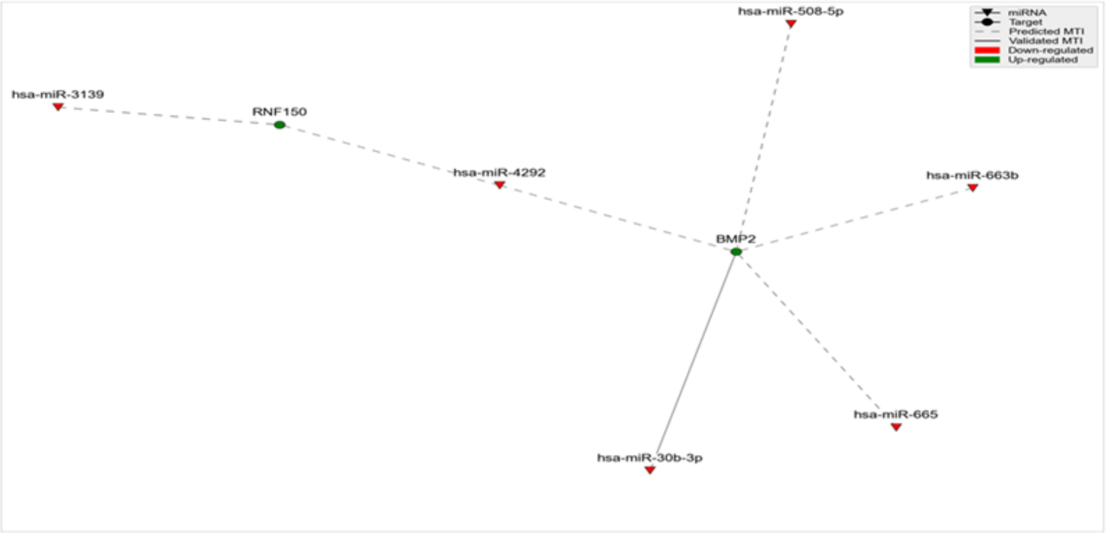

Figure 6: Network of miRNA: target interactions (MTIs). (A) Network of MTIs for genes upregulated in holoclones compared to parental cells. (B) Network of MTIs for genes downregulated in holoclones compared to parental cells. (C) Network of MTIs for genes upregulated in holoclone-derived tumours compared to parental tumours (n=1).

Supplementary Table S1: Integrin α2β1, CD44 and CD133 expression in a panel of prostate cancer cell lines (n=1).

Marker	DU145	PC-3	22Rv1	LNCaP
Integrin$\alpha_2\beta_1$	100	100	100	100
CD44	97.9	96.5	97.7	83
CD133	2.05	3.47	2.3	16.9

Table S2: Genes up- and downregulated in both PC-3 and DU145-derived holoclones. Genes shown are those with a Log2 Fold Change greater than 1.5.

Upregulated				Downregulated
AKR1C1	FOS	MAN1A1	SLC22A23	ASNS
ARRB1	FOSB	MEGF9	SLC2A12	CST6
ARRDC4	GBP2	MX2	SLC5A3	DDIT3
B3GNT7	GPX2	NFKBIZ	SNN	GALNT9
BCL6	HHIPL2	NR4A2	TNFSF10	HOXB9
C10orf10	IFITM1	OAS1	TP53INP1	IL11
CCDC64	KAL1	PCDHGB2	TXNIP	IL24
CEMIP	KCNMB4	PCSK9	ZNF117	STC2
CLU	KITLG	PLCH1		UPP1
CYBRD1	LPCAT1	SCNN1A		VGF
EGR1	LYPD3	SELENBP1		WNT10A

Table S3: Genes up- and downregulated in murine tumours derived from both PC-3 and DU145 holoclones. Genes shown are those with a Log2 Fold Change greater than 1.5.

Upregulated	Downregulated
BMP2	MUC6
RNF150	S100P

Table S4: Shared changes in miRNAs in holoclones derived from PC-3 and DU145 cell lines. miRNAs shown are those with a Log2 fold-change greater than 1.5.

**Downregulated**			**Upregulated**	
hsa-miR-6765-5p	hsa-miR-6800-3p	hsa-miR-1252-3p	hsa-miR-20b-5p	hsa-miR-5192
hsa-miR-619-5p	hsa-miR-7111-3p	hsa-miR-3944-3p	hsa-miR-27a-5p	hsa-miR-8068
hsa-miR-4632-5p	hsa-miR-573	hsa-miR-375	hsa-miR-454-5p	hsa-miR-132-3p
hsa-miR-6127	hsa-miR-6865-3p	hsa-miR-494-5p	hsa-miR-26a-2-3p	hsa-miR-340-5p
hsa-miR-6893-5p	hsa-miR-4706	hsa-miR-5096	hsa-miR-934	hsa-miR-4637
hsa-miR-4783-3p	hsa-miR-4279	hsa-miR-744-3p	hsa-miR-4269	hsa-miR-373-3p
hsa-miR-664a-3p	hsa-miR-146-5p	hsa-miR-653-3p	hsa-miR-3173-5p	hsa-miR-3122
hsa-miR-4766-5p	hsa-let-7f-1-3p	hsa-miR-6752-3p	hsa-miR-6751-5p	hsa-miR-4499
hsa-miR-2682-3p	hsa-miR-876-3p	hsa-miR-340-3p	hsa-miR-190a-5p	hsa-miR-515-3p
hsa-miR-5703	hsa-miR-6090	hsa-miR-10b-5p	hsa-miR-182-3p	hsa-miR-1307-5p
hsa-miR-6759-5p	hsa-miR-6720-3p		hsa-miR-433-5p	hsa-miR-301b-5p
hsa-miR-6793-3p	hsa-miR-665		hsa-miR-5587-3p	hsa-miR-193a-3p
hsa-miR-670-5p	hsa-miR-6794-5p		hsa-miR-7702	hsa-miR-498
hsa-miR-7155-3p	hsa-miR-3940-5p		hsa-miR-128-2-5p	hsa-miR-4655-3p
hsa-miR-3928-3p	hsa-miR-6785-5p		hsa-miR-616-5p	hsa-miR-32-5p
hsa-miR-500a-3p	hsa-miR-6746-3p		hsa-miR-4451	hsa-miR-19b-3p

Table S5: Shared miRNAs that are downregulated in murine tumours derived from PC-3 and DU145 holoclones. miRNAs shown are those with a Log2 fold-change greater than 1.5.

**Downregulated**		
hsa-miR-3174	hsa-miR-6792-5p	hsa-miR- 7109-5p
hsa-miR-508-5p	hsa-miR-940	hsa-miR-4256
hsa-miR-7109-3p	hsa-miR- 6765-3p	hsa-miR-3940-3p
hsa-miR-6769a-3p	hsa-miR-2114-5p	hsa-miR-619-5p
hsa-miR-3617-5p	hsa-miR- 491-5p	hsa-miR-6810-5p
hsa-miR-4302	hsa-miR-6132	hsa-miR-5701
hsa-miR-6813-3p	hsa-miR- 5585-3p	hsa-miR-503-3p
hsa-miR-33a-3p	hsa-miR-663b	hsa-miR-675-5p
hsa-miR-296-3p	hsa-miR- 6872-3p	hsa-miR-744-3p
hsa-miR-1233-5p	hsa-miR-6785-5p	hsa-miR-4284
hsa-miR- 4644	hsa-miR- 937-3p	hsa-miR-1180-3p
hsa-miR-4683	hsa-miR-3651	hsa-miR-8063
hsa-miR-1301-3p	hsa-miR- 4292	hsa-miR-4286
hsa-miR-665	hsa-miR-6068	hsa-miR-4533
hsa-miR-5006-3p	hsa-miR- 4674	hsa-miR-3139
hsa-miR-182-3p	hsa-miR-4801	hsa-miR-4268
hsa-miR-636	hsa-miR-4739	hsa-miR-8074
hsa-miR-381-3p	hsa-miR- 4767	hsa-miR-4638-3p
hsa-miR-23a-5p	hsa-miR-1537-5p	hsa-miR-766-5p
hsa-miR-664a-3p	hsa-miR- 1265	hsa-miR-616-5p
hsa-miR-6859-3p	hsa-miR-4707-5p	hsa-miR-4789-3p
hsa-miR-30b-3p	hsa-miR- 424-3p	hsa-miR-331-3p
hsa-miR-6790-3p	hsa-miR-323a-5p	hsa-miR-5787
hsa-miR-4734	hsa-miR-324-3p	hsa-miR-4787-5p
hsa-miR-4764-5p	hsa-miR- 7108-5p	hsa-miR-4664-5p
hsa-miR-6752-3p	hsa-miR-6727-5p	hsa-miR-3129-3p
hsa-miR-4532	hsa-miR- 4726-5p	hsa-miR-100-3p
hsa-miR-3928-3p	hsa-miR-3607-3p	hsa-miR-3939
hsa-miR-6889-5p	hsa-miR-4690-5p	hsa-miR-6075

Table S6: MTI network data for dysregulated genes in holoclones *vs.* parental cells.

miRNA	Symbol	Status	Source
hsa-let-7f-1-3p	BCL6	Validated	miRWalk
hsa-let-7f-1-3p	CLU	Validated	miRWalk
hsa-let-7f-1-3p	EGR1	Validated	miRWalk
hsa-let-7f-1-3p	KITLG	Validated	miRWalk
hsa-miR-10b-5p	BCL6	Predicted	TargetScan
hsa-miR-10b-5p	HHIPL2	Predicted	miRWalk
hsa-miR-10b-5p	SLC5A3	Predicted	miRWalk
hsa-miR-132-3p	EGR1	Predicted	TargetScan
hsa-miR-132-3p	KITLG	Predicted	TargetScan
hsa-miR-132-3p	MX2	Predicted	miRWalk
hsa-miR-132-3p	NR4A2	Predicted	TargetScan
hsa-miR-146b-5p	EGR1	Predicted	miRTar
hsa-miR-146b-5p	FOS	Validated	miRWalk
hsa-miR-146b-5p	TNFSF10	Validated	miRWalk
hsa-miR-146b-5p	ZNF117	Predicted	miRTar
hsa-miR-146b-5p	ZNF117	Predicted	miRWalk
hsa-miR-182-3p	EGR1	Validated	miRWalk
hsa-miR-190a-5p	TNFSF10	Predicted	miRWalk
hsa-miR-190a-5p	TP53INP1	Predicted	TargetScan
hsa-miR-190a-5p	TP53INP1	Predicted	miRWalk
hsa-miR-193a-3p	BCL6	Predicted	miRTar
hsa-miR-193a-3p	LPCAT1	Predicted	miRTar
hsa-miR-193a-3p	PLCH1	Predicted	miRWalk
hsa-miR-193a-3p	SCNN1A	Predicted	miRTar
hsa-miR-193a-3p	SLC22A23	Predicted	miRTar
hsa-miR-193a-3p	STC2	Predicted	miRTar
hsa-miR-193a-3p	TP53INP1	Predicted	miRTar
hsa-miR-193a-3p	TP53INP1	Predicted	miRWalk
hsa-miR-19b-3p	ARRDC4	Predicted	TargetScan
hsa-miR-19b-3p	ARRDC4	Predicted	miRWalk
hsa-miR-19b-3p	STC2	Predicted	miRWalk
hsa-miR-19b-3p	TP53INP1	Predicted	TargetScan
hsa-miR-19b-3p	WNT10A	Predicted	TargetScan
hsa-miR-20b-5p	CYBRD1	Predicted	TargetScan
hsa-miR-20b-5p	IL11	Predicted	miRWalk
hsa-miR-20b-5p	MEGF9	Predicted	miRWalk
hsa-miR-20b-5p	NR4A2	Predicted	TargetScan
hsa-miR-20b-5p	NR4A2	Predicted	miRWalk
hsa-miR-20b-5p	SLC22A23	Predicted	TargetScan
hsa-miR-20b-5p	SLC5A3	Predicted	TargetScan
hsa-miR-20b-5p	TP53INP1	Predicted	TargetScan
hsa-miR-20b-5p	TP53INP1	Predicted	miRWalk
hsa-miR-20b-5p	TXNIP	Predicted	TargetScan
hsa-miR-20b-5p	TXNIP	Predicted	miRWalk
hsa-miR-27a-5p	KITLG	Validated	miRWalk
hsa-miR-3122	KAL1	Predicted	miRTar
hsa-miR-3122	KITLG	Predicted	miRTar
hsa-miR-3122	MX2	Predicted	miRTar
hsa-miR-3122	SLC2A12	Predicted	miRTar

miRNA	Symbol	Status	Source
hsa-miR-3122	UPP1	Predicted	miRTar
hsa-miR-32-5p	ARRDC4	Predicted	TargetScan
hsa-miR-32-5p	ARRDC4	Validated	miRTarBase
hsa-miR-32-5p	ARRDC4	Predicted	miRWalk
hsa-miR-32-5p	SNN	Predicted	TargetScan
hsa-miR-32-5p	SNN	Predicted	miRWalk
hsa-miR-340-5p	CCDC64	Predicted	TargetScan
hsa-miR-340-5p	EGR1	Predicted	TargetScan
hsa-miR-340-5p	LPCAT1	Predicted	TargetScan
hsa-miR-340-5p	LPCAT1	Validated	miRTarBase
hsa-miR-340-5p	NR4A2	Predicted	TargetScan
hsa-miR-340-5p	SLC5A3	Predicted	TargetScan
hsa-miR-340-5p	TNFSF10	Predicted	miRWalk
hsa-miR-373-3p	BCL6	Predicted	TargetScan
hsa-miR-373-3p	CST6	Predicted	miRWalk
hsa-miR-373-3p	CYBRD1	Predicted	TargetScan
hsa-miR-373-3p	NR4A2	Predicted	TargetScan
hsa-miR-373-3p	NR4A2	Predicted	miRWalk
hsa-miR-373-3p	SLC22A23	Predicted	TargetScan
hsa-miR-373-3p	SLC2A12	Predicted	miRWalk
hsa-miR-373-3p	TP53INP1	Predicted	TargetScan
hsa-miR-373-3p	TXNIP	Predicted	TargetScan
hsa-miR-373-3p	TXNIP	Validated	miRTarBase
hsa-miR-373-3p	TXNIP	Validated	miRWalk
hsa-miR-373-3p	ZNF117	Predicted	miRWalk
hsa-miR-375	ARRB1	Predicted	TargetScan
hsa-miR-375	B3GNT7	Predicted	miRTar
hsa-miR-375	LPCAT1	Validated	miRTarBase
hsa-miR-375	LYPD3	Predicted	miRTar
hsa-miR-375	STC2	Predicted	miRTar
hsa-miR-4269	ARRDC4	Predicted	miRTar
hsa-miR-4269	B3GNT7	Predicted	miRTar
hsa-miR-4269	FOS	Predicted	miRTar
hsa-miR-4269	IFITM1	Predicted	miRTar
hsa-miR-4269	IL24	Predicted	miRTar
hsa-miR-4269	KAL1	Predicted	miRTar
hsa-miR-4269	MAN1A1	Predicted	miRTar
hsa-miR-4269	MEGF9	Predicted	miRTar
hsa-miR-4269	NR4A2	Predicted	miRTar
hsa-miR-4269	PCSK9	Predicted	miRTar
hsa-miR-4269	PLCH1	Predicted	miRTar
hsa-miR-4269	SELENBP1	Predicted	miRTar
hsa-miR-4269	SLC22A23	Predicted	miRTar
hsa-miR-4269	SNN	Predicted	miRTar
hsa-miR-4269	STC2	Predicted	miRTar
hsa-miR-4269	ZNF117	Predicted	miRTar
hsa-miR-4279	ASNS	Predicted	miRTar
hsa-miR-4279	BCL6	Predicted	miRTar
hsa-miR-4279	C10orf10	Predicted	miRTar
hsa-miR-4279	DDIT3	Predicted	miRTar
hsa-miR-4279	EGR1	Predicted	miRTar

miRNA	Symbol	Status	Source
hsa-miR-4279	FOSB	Predicted	miRTar
hsa-miR-4279	LPCAT1	Predicted	miRTar
hsa-miR-4279	MAN1A1	Predicted	miRTar
hsa-miR-4279	MEGF9	Predicted	miRTar
hsa-miR-4279	MX2	Predicted	miRTar
hsa-miR-4279	PCSK9	Predicted	miRTar
hsa-miR-4279	SCNN1A	Predicted	miRTar
hsa-miR-4279	SLC22A23	Predicted	miRTar
hsa-miR-4279	SNN	Predicted	miRTar
hsa-miR-4279	STC2	Predicted	miRTar
hsa-miR-4279	TP53INP1	Predicted	miRTar
hsa-miR-4279	VGF	Predicted	miRTar
hsa-miR-498	FOS	Predicted	miRWalk
hsa-miR-498	KAL1	Predicted	miRTar
hsa-miR-498	SLC22A23	Predicted	miRTar
hsa-miR-515-3p	BCL6	Predicted	miRTar
hsa-miR-515-3p	CYBRD1	Predicted	miRTar
hsa-miR-515-3p	KITLG	Predicted	miRTar
hsa-miR-515-3p	LPCAT1	Predicted	miRTar
hsa-miR-515-3p	MX2	Predicted	miRTar
hsa-miR-515-3p	NFKBIZ	Predicted	miRTar
hsa-miR-515-3p	OAS1	Predicted	miRTar
hsa-miR-515-3p	SCNN1A	Predicted	miRTar
hsa-miR-515-3p	SELENBP1	Predicted	miRTar
hsa-miR-515-3p	SNN	Predicted	miRTar
hsa-miR-515-3p	TP53INP1	Predicted	miRTar
hsa-miR-515-3p	VGF	Predicted	miRTar
hsa-miR-515-3p	WNT10A	Predicted	miRWalk
hsa-miR-573	CLU	Predicted	miRTar
hsa-miR-573	CST6	Predicted	miRWalk
hsa-miR-573	FOS	Predicted	miRWalk
hsa-miR-573	GBP2	Predicted	miRTar
hsa-miR-573	MEGF9	Predicted	miRTar
hsa-miR-573	NFKBIZ	Predicted	miRTar
hsa-miR-573	NFKBIZ	Predicted	miRWalk
hsa-miR-573	PCSK9	Predicted	miRTar
hsa-miR-573	PLCH1	Predicted	miRTar
hsa-miR-573	SLC22A23	Predicted	miRTar
hsa-miR-573	TNFSF10	Predicted	miRWalk
hsa-miR-573	ZNF117	Predicted	miRTar
hsa-miR-665	ASNS	Predicted	miRTar
hsa-miR-665	BCL6	Predicted	miRTar
hsa-miR-665	CST6	Predicted	miRTar
hsa-miR-665	CYBRD1	Predicted	miRTar
hsa-miR-665	DDIT3	Predicted	miRTar
hsa-miR-665	FOSB	Predicted	miRTar
hsa-miR-665	GALNT9	Predicted	miRTar
hsa-miR-665	HOXB9	Predicted	miRTar
hsa-miR-665	IL11	Predicted	miRTar
hsa-miR-665	KAL1	Predicted	miRTar
hsa-miR-665	LPCAT1	Predicted	miRTar

miRNA	Symbol	Status	Source
hsa-miR-665	MAN1A1	Predicted	miRTar
hsa-miR-665	MEGF9	Predicted	miRTar
hsa-miR-665	MX2	Predicted	miRTar
hsa-miR-665	OAS1	Predicted	miRTar
hsa-miR-665	PCSK9	Predicted	miRTar
hsa-miR-665	SCNN1A	Predicted	miRTar
hsa-miR-665	SELENBP1	Predicted	miRTar
hsa-miR-665	SLC22A23	Predicted	miRTar
hsa-miR-665	SLC2A12	Predicted	miRTar
hsa-miR-665	SNN	Predicted	miRTar
hsa-miR-665	SNN	Predicted	miRWalk
hsa-miR-665	STC2	Predicted	miRTar
hsa-miR-665	TNFSF10	Predicted	miRTar
hsa-miR-665	TXNIP	Predicted	miRTar
hsa-miR-665	VGF	Predicted	miRTar
hsa-miR-665	WNT10A	Predicted	miRTar
hsa-miR-665	ZNF117	Predicted	miRTar
hsa-miR-744-3p	KCNMB4	Predicted	miRWalk
hsa-miR-876-3p	HOXB9	Predicted	miRWalk
hsa-miR-934	DDIT3	Predicted	miRWalk

Table S7: MTI network data for dysregulated genes in murine tumours (holoclones *vs.* parental).

miRNA	Symbol	Status	Source
hsa-miR-1265	MUC6	Predicted	miRTar
hsa-miR-296-3p	MUC6	Predicted	miRTar
hsa-miR-30b-3p	BMP2	Validated	miRWalk
hsa-miR-3139	MUC6	Predicted	miRTar
hsa-miR-3139	RNF150	Predicted	TargetScan
hsa-miR-3139	RNF150	Predicted	miRTar
hsa-miR-324-3p	MUC6	Predicted	miRTar
hsa-miR-331-3p	MUC6	Predicted	miRWalk
hsa-miR-4256	MUC6	Predicted	miRTar
hsa-miR-4268	MUC6	Predicted	miRTar
hsa-miR-4284	MUC6	Predicted	miRTar
hsa-miR-4292	BMP2	Predicted	miRTar
hsa-miR-4292	RNF150	Predicted	miRTar
hsa-miR-4302	MUC6	Predicted	miRTar
hsa-miR-491-5p	MUC6	Predicted	miRTar
hsa-miR-508-5p	BMP2	Predicted	miRTar
hsa-miR-508-5p	MUC6	Predicted	miRTar
hsa-miR-628-5p	BMP2	Predicted	miRTar
hsa-miR-628-5p	RNF150	Predicted	miRWalk
hsa-miR-663b	BMP2	Predicted	miRTar
hsa-miR-663b	MUC6	Predicted	miRTar
hsa-miR-665	BMP2	Predicted	miRTar
hsa-miR-665	MUC6	Predicted	miRTar
hsa-miR-665	MUC6	Predicted	miRWalk
hsa-miR-665	S100P	Predicted	miRTar
hsa-miR-940	MUC6	Predicted	miRTar
